# LC-QToF chemical profiling of *Euphorbia grantii* Oliv. and its potential to inhibit LPS-induced lung inflammation in rats via the NF-κB, CY450P2E1, and P38 MAPK14 pathways

**DOI:** 10.1007/s10787-023-01298-7

**Published:** 2023-08-12

**Authors:** Mai Hussin Radi, Riham A. El-Shiekh, Amany Mohammed Hegab, Shirley Ragae Henry, Bharathi Avula, Kumar Katragunta, Ikhlas A. Khan, Ali M. El-Halawany, Essam Abdel-Sattar

**Affiliations:** 1 Herbal Department, Egyptian Drug Authority (EDA), Giza, Egypt; 2 Pharmacognosy Department, Faculty of Pharmacy, Cairo University, Cairo, 11562 Egypt; 3 Developmental Pharmacology Department, Egyptian Drug Authority (EDA), Giza, Egypt; 4 Histopathology Department, Egyptian Drug Authority (EDA), Giza, Egypt; 5 School of Pharmacy, National Center for Natural Products Research, University of Mississippi, University, MS 38677 USA; 6 Division of Pharmacognosy, Department of BioMolecular Sciences, School of Pharmacy, University of Mississippi, University, MS 38677 USA

**Keywords:** *Euphorbia grantii*, Lipopolysaccharide, Anti-inflammatory, Acute lung injury, LC-DAD-QToF chemical profiling, In vitro and in vivo

## Abstract

Acute lung injury (ALI) is a life-threatening syndrome that causes high morbidity and mortality worldwide. The aerial parts of *Euphorbia grantii* Oliv. were extracted with methanol to give a total methanolic extract (TME), which was further fractionated into dichloromethane (DCMF) and the remaining mother liquor (MLF) fractions. Biological guided anti-inflammatory assays in vitro revealed that the DCMF showed the highest activity (IC_50_ 6.9 ± 0.2 μg/mL and 0.29 ± 0.01 μg/mL) compared to*.* celecoxib (IC_50_ of 88.0 ± 1 μg/mL and 0.30 ± 0.01 μg/mL) on COX-1 and COX-2, respectively. Additionally, anti-LOX activity was IC_50_ = 24.0 ± 2.5 μg/mL vs. zileuton with IC_50_ of 40.0 ± 0.5 μg/mL. LC-DAD-QToF analysis of TME and the active DCMF resulted in the tentative identification and characterization of 56 phytochemical compounds, where the diterpenes were the dominated metabolites. An LPS-induced inflammatory model of ALI (10 mg/kg i.p) was used to assess the anti-inflammatory potential of DCMF in vivo at dose of 200 mg/kg and 300 mg/kg compared to dexamethasone (5 mg/kg i.p). Our treatments significantly reduced the pro-inflammatory cytokines (TNF-α, IL-1, IL-6, and MPO), increased the activity of antioxidant enzymes (SOD, CAT, and GSH), decreased the activity of oxidative stress enzyme (MDA), and reduced the expression of inflammatory genes (p38.MAPK14 and CY450P2E1). The western blotting of NF-κB p65 in lung tissues was inhibited after orally administration of the DCMF. Histopathological study of the lung tissues, scoring, and immunohistochemistry of transforming growth factor-beta 1 (TGF-β1) were also assessed. In both dose regimens, DCMF of *E. grantii* prevented further lung damage and reduced the side effects of LPS on acute lung tissue injury.

**Supplementary Information:**

The online version contains supplementary material available at 10.1007/s10787-023-01298-7.

## Introduction

Inflammation is a complex reaction that involves interactions between antigen-presenting cells (i.e., APCs), monocytes, and activated lymphocytes, which then differentiate into macrophages. During this process, a large number of cytokines are released (Latruffe 2017). Inflammation is also an early response of vascular tissues to infection, injuries, and harmful stimuli such as pathogens and irritants. Furthermore, inflammation is involved in non-specific immune responses that aim to neutralize invaders, repair damaged cells, and initiate the healing processes (Ferrero-Miliani et al. 2007).

Acute lung injury (ALI) and acute respiratory distress syndrome (ARDS) are acute respiratory failure syndromes characterized by severe pulmonary edema, neutrophil accumulation, and hypoxemia in the lungs (Luh and Chiang 2007). The incidence of ALI/ARDS is high and is associated with marked mortality and morbidity (Zhang et al. 2009). ALI/ARDS is the most common reason of severe respiratory failure caused by damage to the alveoli and capillary barrier (Lee et al. 2021). ARDS occurs with 75% of cases categorized as moderate or severe conditions (Matthay et al. 2019). The fatality rates from mild to severe ARDS remain of about 27–45%, which is considerably higher than for other acute/chronic diseases as pneumonia, asthma, and myocardial infarction (Diamond et al. 2020). SARS-CoV-2 at the heart of the COVID-19 pandemic has worsened the threat of ARDS from a major healthcare alarm to a global crisis worldwide (Li and Ma 2020). Severe inflammation of the airway caused by inhalation of infectious substances into the bronchi is very important in the pathogenesis of ARDS (Han and Mallampalli 2015). ARDS is an acute lung disease with numerous causes that involves various cellular factors alongside the pathogenic growth and results in multiple types of destruction to the tissues (Matthay and Zemans 2011; Sharp et al. 2015). Only symptomatic relief drugs are currently available, as inhaled nitric oxide or steroidal and nonsteroidal anti-inflammatory drugs (NSAIDs) (Gebistorf et al. 2016; Khilnani and Hadda 2011). Numerous side effects are reported for their uses as hypertension, gastrointestinal perforation, peptic ulcers, hemorrhage, gastrointestinal distress, and inflammation (Ho et al. 2020). Though, the efficacy and safety of these therapies remain inadequate and there is a high medical need for a ultimate treatment for ARDS (Patel et al. 2018).

Lipopolysaccharide (LPS), commonly observed in the cell wall of gram-negative bacteria, is known to induce inflammation. LPS-induced acute lung injury (ALI), an animal model of severe pulmonary inflammation, is widely used to study ARDS (Rittirsch et al. 2008; Qi et al. 2017). Injection of LPS stimulates macrophages to produce the proinflammatory cytokines such as TNF-α, IL-6, IL-10, and IL-1β, which induce the infiltration and activation of neutrophils (Rittirsch et al. 2008; Chen et al. 2020; de Souza et al. 2017). Stimulated neutrophils next produce a large amount of reactive oxygen species (ROS) and cytokines, further worsen severe lung damage through damaging inflammatory responses (Potey et al. 2019). LPS stimulates the release and expression of inflammatory cytokines, resulting in an acute inflammatory response by activating the Toll-like receptor 4 (TLR4)-dependent pathway, leading to the rapid releases of pro-inflammatory cytokines (Al-Rikabi et al. 2020). Inflammation and oxidative stress play a significant role in the pathogenic process, which is generated by the innate immune response to LPS-induced acute tissue injury (Zhu et al. 2015). Although corticosteroids remain the mainstay of ALI treatments, they result in high toxicity and side effects (Sudhakaran et al. 2022). Therefore, it is essential to incorporate a natural anti-inflammatory agent into medication therapies to achieve increased pharmacological effects with minimal side effects. These are as well the pathogenic attributes of infection with SARS-CoV-2 (Delgado-Roche and Mesta 2020).

We assumed that herbs and/or their extracts could be used as effective candidate for ARDS since they contain multiple compounds with health benefits. Many therapeutic extracts have been broadly documented for their safety and efficacy, with a variety of chemical compounds that may regulate the cellular or biochemical pathways involved in the pathogenesis of ARDS (Chen et al. 2018; Dai et al. 2014; Han et al. 2013; Huang et al. 2018). The potential anti-inflammatory effects of Euphorbia plants were documented in the literature (Abo-Dola and Lutfi 2016).

The genus *Euphorbia* is the largest in the spurge family, comprising about 2000 species. Some species of the genus have been reported as medicinal plants for the treatment of gonorrhea, intestinal parasites, migraine, and skin diseases. Diterpenoids are the major phytoconstituents with many different core frameworks such as tiglianes, jatrophanes, myrsinols, lathyranes, ingenanes, etc. The triterpene alcohols in the latex of *Euphorbia* species have been used as chemotaxonomic markers. Additionally, sesquiterpenoids, phloroacetophenones, glycerols, cerebrosides, flavonoids, and steroids were also found (Shi et al. 2008; Radi et al. 2023a, b).

Euphorbia diterpenes are promising compounds for multidrug resistance reversal abilities and showed the ability to act as anti-inflammatory agents both in vivo and in vitro (Kemboi et al. 2021; Wei et al. 2019). To explore more species of Euphorbia as anti-inflammatory agent, the authors investigated the potential anti-inflammatory effects of *Euphorbia grantii* Oliv. for the first time. The aim of our study was to document the anti-inflammatory actions of *Euphorbia grantii* Oliv. through biological-guided fractionation using in vitro methods. The total extract and the active fraction were investigated using LC/MS technique to identify the metabolites responsible for the activity. Additionally, the active fraction was tested in vivo using a rat model to investigate its protective effects in LPS-induced ALI. It is worth noting that, upon reviewing the current literature, no data regarding the chemical profile of *E. grantii* aerial parts were found.

## Materials and methods

### Chemicals

Biochem Company (Cairo, Egypt) supplied solvents used in the extraction and fractionation procedures and were of analytical grade. Lipopolysaccharide (LPS), dexamethasone (DEX), and phosphate-buffered saline were purchased from Sigma Aldrich (St. Louis, MO, USA).

### Plant material

*Euphorbia grantii* Oliv. aerial parts were collected from El-Orman Botanical Garden, Giza, Egypt in January 2020. The plant material was identified by Ms. Therese Labib, a Botanical Specialist and Consultant at El-Orman Botanic Garden. A voucher specimen (No. 4.01.2023 I) was deposited at the herbarium of Pharmacognosy, Faculty of Pharmacy, Cairo University, Cairo, Egypt. The aerial parts were washed with tap water to remove debris, dust, and solid materials, and then left to dry in shade. The dried aerial parts were powdered and sieved to 80 mesh, and the powder was stored in a sealed container until use.

### Extraction and fractionation

Ten kilograms of fresh plant material were dried to give 600 g of the dried plant powder. The air-dried plant material was extracted by methanol (5 × 5 L) with an extraction temperature of 50 °C, an extraction time of 3 days and 100% methanol as solvent. The collected extract was filtered and evaporated under reduced pressure to give 81.5 g of total methanolic extract (TME). A portion of the TME (50 g) was fractionated in separating funnel using liquid–liquid fractionation method and methanol, water, and dichloromethane (DCM) in a ratio of 1:1:1 (3 X 1 L). The content can settle, and the bottom of the separating funnel opened to collect the organic DCM layer which was evaporated and resulted in a dichloromethane fraction (DCMF, 30 g) and the upper layer was collected and evaporated to get the remaining mother liquor fraction (MLF, 22 g).

### Cyclooxygenases (COX) and lipoxygenase (LOX) inhibitory assay

#### COX-1 and COX-2 inhibition assay

The extract and its respective fractions were dissolved in 100% DMSO to prepare a stock concentration of 5 mg/ml. The samples were tested in triplicates at serial dilutions of (0.1–100 μg/ml). The inhibitory COX activity was assayed calorimetrically, as previously described method (George et al. 2014) and using Cayman colorimetric COX (ovine) inhibitor screening assay kit (Cayman Chemical Company, MI, USA), according to the manufacturer’s instructions. Celecoxib was run as the positive control for inhibition of COX-1 and COX-2. A volume of 10 μl each of test samples and vehicle were diluted to 20 μl with 0.1 M Tris–HCl (pH 8.0) and pre-incubated with the enzyme at 37 °C for 15 min before the addition of arachidonic acid. Then, the reaction was initiated by addition of 10 μl 10 mM arachidonic acid after that the reaction mixture was incubated at 37 °C for another 2 min. Reaction was terminated by addition of 50 μl 1 N HCl and saturated stannous chloride. Assays were performed using 100 U of ovine COX-1 and COX-2. An aliquot is removed and the prostanoid produced is quantified spectrophotometrically at 405 nm. The results were expressed in IC_50_ (μg/mL).

#### 5-Lipoxygenase (5-LOX) inhibitory assay

The assay was carried out calorimetrically (Nishida et al. 2014), using LOX inhibitor screening assay kit (Cayman Chemical Company, MI., USA) according to the manufacturer’s instructions. The samples were tested in triplicates at 10 and 100 μg/ml using. Zileuton was used as the positive control. A volume of 10 μl each of test samples and vehicle were pre-incubated with 90 μl 5-LOX enzyme in a 96-well plate. The reaction was initiated by addition of 10 μl 1 mM arachidonic acid and the plate was shaken for 5 min. Then, 100 μl of chromogen from the test kit was added to stop enzymic reaction and for color development. The plate was placed on a shaker for another 5 min and absorbance at 490 nm was measured using microplate reader. The results were expressed in IC_50_ (μg/mL).

### Liquid chromatography diode array detector-quadrupole time-of-flight mass spectrometry (LC-DAD-QToF) analysis

Samples TME and DCMF were prepared in HPLC-grade methanol, filtered, and placed into sealed LC vials prior to analysis. The samples were then subjected to LC-DAD-QToF analysis. The liquid chromatographic equipment used was an Agilent Series 1290, and the separation was accomplished using an Acquity UPLC™ HSS C18 column (100 mm × 2.1 mm I.D., 1.8 µm). The injection volume was 2 μL, the mobile phase consisted of water with 0.1% formic acid (A) and acetonitrile with 0.1% formic acid (B) at a flow rate of 0.23 mL/min. Analysis was performed using the following gradient elution: 9 5% A and 5% B to 100% B in 35 min. Each run was followed by a 3 min wash with 100% B and an equilibration period of 5 min with 95% A and 5% B. The mass spectrometric analysis was achieved with a QToF-MS–MS (Model #G6545B, Agilent Technologies, Santa Clara, CA, USA) equipped with an ESI source with Jet Stream technology using the following parameters: drying gas (N_2_) flow rate, 13 L/min; drying gas temperature, 300 °C; nebulizer pressure, 20 psig, sheath gas temperature, 400 °C; sheath gas flow, 12 L/min; capillary voltage, 4000 V; nozzle voltage, 0 V; skimmer, 65 V; Oct RF V, 750 V; and fragmentor voltage, 150 V. Agilent Mass Hunter Acquisition Software Ver. A.10.1 was used to control all data acquisition and analysis procedures, and Mass Hunter Qualitative Analysis Software Ver. B.10.00 was used to process the data. Each sample was evaluated in positive mode spanning the *m/z* 50–1700 range as well as the extended dynamic range (flight time to *m/z* 1700 at 2 GHz acquisition rate). In positive ion mode, accurate mass measurements were accomplished by employing reference ion correction by using references masses at *m/z* 121.0509 (protonated purine) and 922.0098 (protonated hexakis (1H, 1H, 3H-tetrafluoropropoxy) phosphazine or HP-921), while in negative ion mode at *m/z* 112.9856 (deprotonated trifluoroacetic acid-TFA) and 1033.9881 (TFA adducted HP-921) were used. Samples were examined in all-ion MS–MS mode, with experiment 1 using a collision energy of zero and experiment 2 using a collision energy of 45 eV. Each spectrum confirmed the compounds.

### In vivo anti-inflammatory assay

#### Acute study (LD_50_)

An acute study was conducted prior to the main study to determine the LD_50_ dose of the TME. Twenty healthy male albino mice, weighing 20–22 g were randomly divided into four groups (5 per group) and administered four oral doses (500 mg/kg, 1000 mg/kg, 1500 mg/kg, 3000 mg/kg) under suitable environmental conditions (temperature 20–25 °C, humidity 40–50%, light cycle 12 h light: 12 h dark, and ad libitum food and drinking water). All animals were observed for 24 h to calculate the LD_50_ (Ahmed 2015). No mortality was observed in any of the plant extract doses, indicating a high margin of safety for the extracts. The experiments were carried out according to the National Organization for Drug Control & Research Ethics Committees, Approval No.: (#NODCAR/1/2/2023).

#### Experimental design

Thirty-five free pathogenic Sprague–Dawley male albino rats (100–120 g) were obtained from the Egyptian Drug Authority (EDA) animal house. Randomly, the animals were divided into seven groups, (each 5 rats) as follows: Group I: normal control group, Group II: positive control group on the 7th day only received LPS (Lipopolysaccharides) at a dose of 10 mg/kg bw. i.p. suspended in sterile saline solution 0.9% (Ijinu et al. 2022), Group III dexamethasone positive control (5 mg/kg i.p.) (Murillo-Cuesta et al. 2023) for 7 days followed by LPS single inflammatory dose model (10 mg/kg b. w i.p.). On the 7th day, 1 h after the last dexamethasone dose. Groups IV and V received the DCMF orally at doses of 200 and 300 mg/kg (1/10 LD50) for seven consecutive days followed by LPS 10 mg/kg b.wt. i.p) on the 7th day, 1 h after plant extract administration, and Groups VI and VII received the DCMF orally at doses of 200 and 300 mg/kg. All animals were anesthetized to obtain blood in heparinized capillary tubes for pro-inflammatory cytokines (TNF-α, IL-1, IL-6, and MPO) ELISA examination in addition to lung tissues for determination of antioxidant enzymes (SOD, Catalase, GSH and MDA), real-time PCR (p38.MAPK14 and CY450P2E1), western blot (NF-κB p65), histological lung tissue scoring and immunohistochemistry examination (TGF-β1).

#### ELISA Assay Principles in Serum

Rat TNF-α (Catalog No: MBS2507393 96 T/48 T/24 T), IL-6 (Catalog No: MBS355410), IL-1β (Catalog no. MBS825017), MPO (Catalog No. MBS2019590) ELISA kits were obtained from MyBiosource, San Diego, USA**.**

#### Gene expression examination

The primers supplied by Metabion (Qiagen, Germany, GmbH) were listed in the Table 1. Primers were utilized in a 25- µl reaction containing 12.5 µl of the 2 × QuantiTect SYBR Green PCR Master Mix (StrateGene® MX3005P, Qiagen, Germany), 0.25 µl of Revert Aid reverse transcriptase (200 U/µL) (Thermo Fisher, Germany), 0.5 µl of each primer of 20 pmol concentration, 8.25 µl of water, and 3 µl of RNA template. The experiment was carried out on a Strata Gene MX3005P real-time PCR machine (Qiagen, Germany, GmbH). Amplification curves and Ct values were determined by the Strata gene MX3005P software, version 3 (Agilent, California, USA). To estimate the variation of gene expression on the RNA of the different samples, the CT of each sample was compared with that of the positive control group according to the "ΔΔCT" method stated by Yuan et al. (2006).

**Table 1:** Primers sequences, target genes, amplicon sizes, and cycling conditions for SYBR green rt-PCR

Target gene	Primers sequences	References
*ß-actin*		Raff et al. (1997)
Forward	5′ CCTCCTGAGCGCAAGTACTCT	
Reverse	3′ GCTCAGTAACAGTCCGCCTAGAA	
*P38.MAPK14*		Wang et al. (2015)
Forward	5ʹ CGAGCGATACCAGAACCTGT	
Reverse	3ʹ GCGTGAATGATGGACTGAAA	
*CY450P2E1*		Wang et al. (2017)
Forward	5ʹ CTCCTCGTCATATCCATCTG	
Reverse	3ʹ GCAGCCAATCAGAAATGTGG	

#### Western blot examination

Samples were separated on a polyacrylamide gel; the procedure was abbreviated as SDS-PAGE, or Sodium Dodecyl Sulfate Polyacrylamide Gel Electrophoresis, a standard technique for separating proteins according to their molecular weight. Polyacrylamide gels were performed using the TGX Stain-FreeTM Fast Cast™ Acrylamide Kit (SDS-PAGE), which was provided by Bio-Rad Laboratories, inc Cat # 161–0181(Alexa Fluor, USA). Blocking the membrane, the membrane was blocked in tris-buffered saline with Tween 20 (TBST) buffer and 3% bovine serum albumin (BSA) at room temperature for 1 h. Incubation with the primary antibody: primary antibodies Santa Cruz Biotechnology, Inc. (Alexa Fluor, Oregon, USA), NF-κB p65 (F-6): sc-8008 The primary antibody was diluted in TBST according to the manufacturer's instructions. Incubation was done overnight in each primary antibody solution, against the blotted target protein at 4 °C. The blot was rinsed 3–5 times for 5 min with TBST. Incubation was done in the HRP-conjugated secondary antibody solution (Goat anti-rabbit IgG-HRP-1 HRP-1 mg Goat mab, Novus Biologicals) against the blotted target protein for 1 h. at room temperature. The blot was rinsed 3–5 times for 5 min with TBST. Imaging and data analysis quantitation: The chemiluminescent substrate (Clarity TM Western ECL substrate, Bio-Rad cat# 170-5060) was applied to the blot according to the manufacturer’s recommendation.

#### Histopathological study

Lung tissues were collected and fixed in 10% neutral buffered formaldehyde for 24 h to stabilize and fix tissue proteins. Dehydration, clearing, and paraffin infiltration and embedding were performed, and then wax blocks were sectioned by microtome into 5 µ sections and stained with Hematoxylin and Eosin Stain. Hematoxylin staining was performed according Ehrlich's Hematoxylin (Ehrlich 1886) and eosin staining was done according to Avwioro (2011). Anti-Transforming Growth Factor-β (TGF-β) antibody immunohistochemistry stain (Arigo Biolaboratories, ARG 56429) was also performed, and Immuno-histochemical staining was performed according to Bajracharya et al. (2017). The same position of the lungs in five mice was observed through a microscope, and the histological lesion of the lung was evaluated through the incidence of congestion, inflammation, and hemorrhage, severity scoring of neutrophil infiltrates, and proportion of airspace areas. The level of severity was judged from – to +++, representing none to severe. The airspace proportion is the ratio of the airspace area and the total area of one view under 400 × magnifications (Wang et al. 2018). According to Xue et al. (2011) & Ai-Li Huang et al. (2014): the semi-quantitative criterion for TGF-β1 staining intensity was estimated using a four-tiered scoring system: negative (−), no staining at all; weak (+), weak staining regardless of positive cell percentages or moderate staining of ≤ 30% of cells; moderate (++), moderate staining of > 30% of cells or strong staining of ≤ 50% of cells; strong (+++), strong staining of > 50% of cells. Low expression of TGF-β1 was defined as (-) or ( +), and High expression was defined as (++) or (+++).

#### Statistical analysis

The present data were analyzed by SPSS version 20.0 (IBM, NY, USA). The measurement data were represented as the mean value ± standard error of mean (SEM). Differences between multiple groups were analyzed via one-way ANOVA followed by Tukey’s post hoc test after testing for normality (by the Shapiro–Wilk test). *P* < 0.05 denoted statistical significance.

## Results

### Cyclooxygenases and lipoxygenase inhibitory activities

As shown in Table 2, among the tested samples the DCMF showed the highest anti-inflammatory activity in vitro. The IC_50_ of DCMF showed better results than that of the standard drug celecoxib against COX-1 with IC_50_ of 6.9 ± 0.2 μg/mL and 88.0 ± 1 μg/mL, respectively). The anti-COX-2 activity of DCMF 0.29 ± 0.01 μg/mL vs. celecoxib with IC_50_ of 0.30 ± 0.01 μg/mL. Additionally, anti-LOX activity of DCMF 24 ± 2.5 μg/mL *vs.* zileuton with IC_50_ of 40.0 ± 0.5 μg/mL.

**Table 2:** Cyclooxygenases and lipoxygenase inhibitory activity of TIME, DCMF, and MLF (IC_50_, μg/mL)

Samples	COX-1	COX-2	LOX
TME	19.8 ± 1.3	0.5 ± 0.03	31 ± 3.1
DCMF	6.9 ± 0.2	0.29 ± 0.01	24 ± 2.5
MLF	51.0 ± 3.4	4.0 ± 0.9	34 ± 3.2
Celecoxib	88.0 ± 1	0.30 ± 0.01	–
Zileuton	–	–	40.0 ± 0.5

### LC-DAD-QToF-MS analysis

**Fig. 1:**
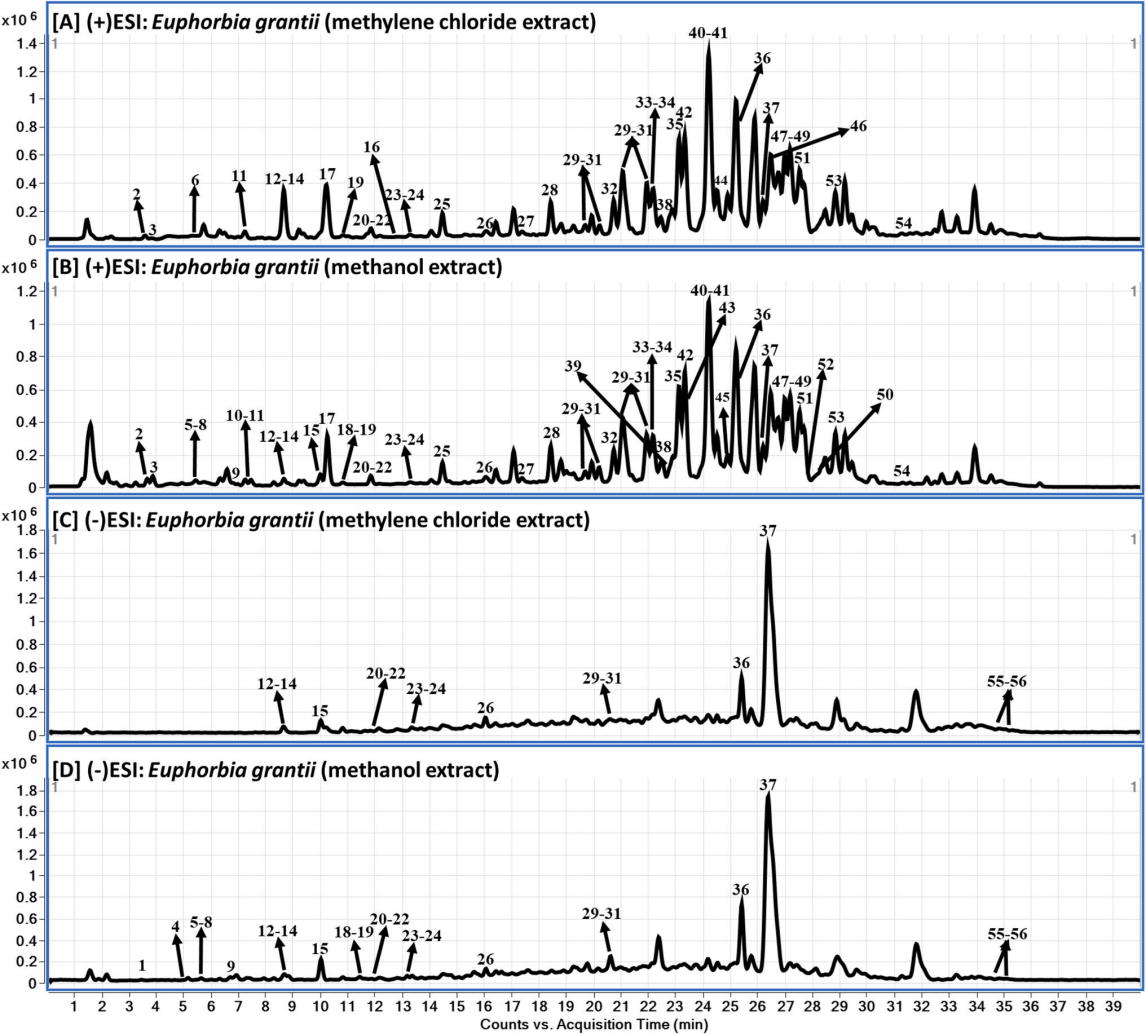
LC-DAD-QToF chromatograms of *Euphorbia grantii*: **A**-**D** QToF-MS Base Peak chromatograms of methylene chloride and methanol extracts in positive and negative modes, respectively

**Fig. 2:**
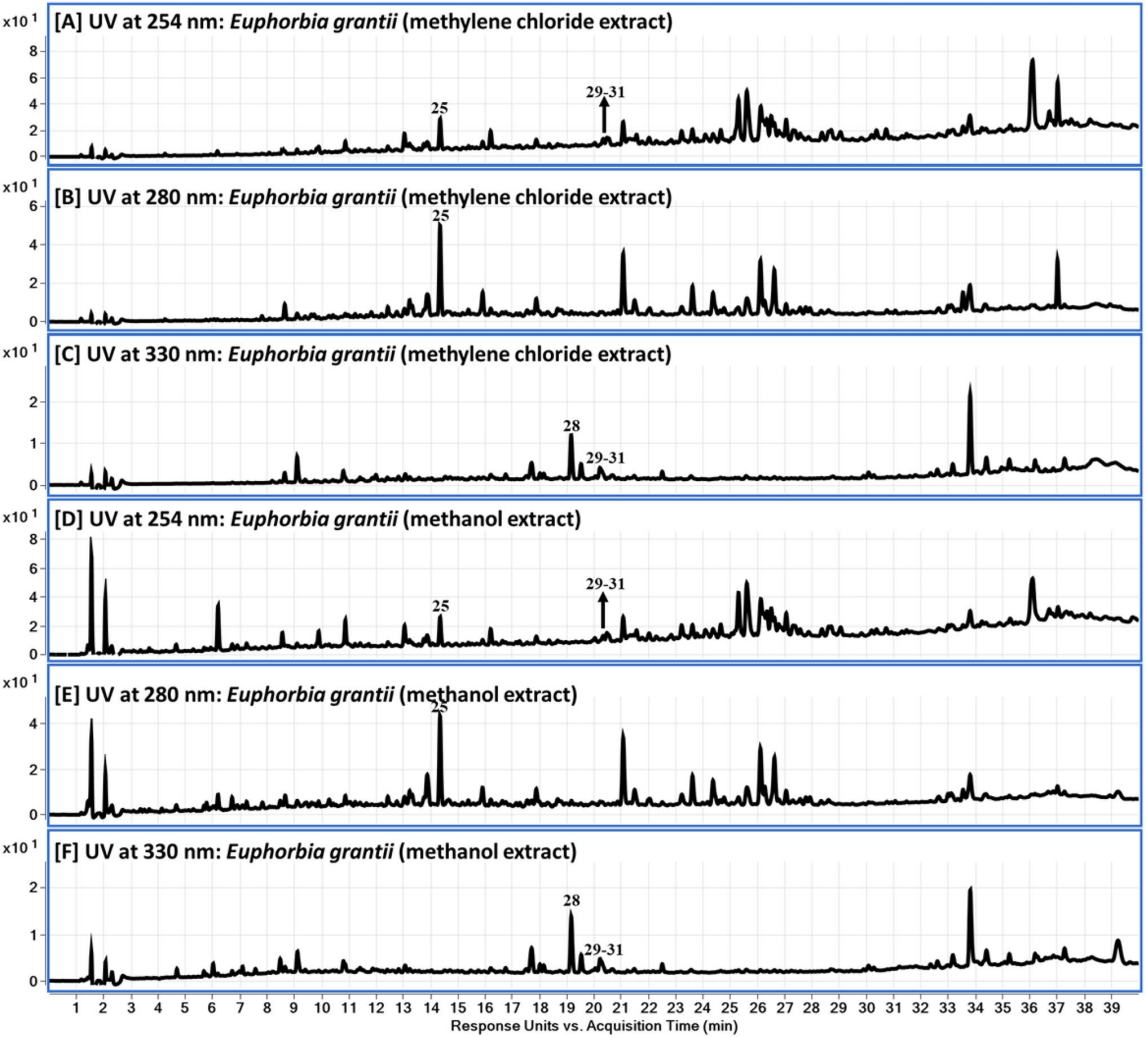
LC-DAD-QToF chromatograms of *Euphorbia grantii*: **A-F** DAD chromatograms for methylene chloride and methanol extracts at 254, 280 and 330 nm

The TME and the active DCMF metabolite profiling were investigated using liquid chromatography diode array detector-quadrupole time-of-flight mass spectrometry (LC-DAD-QToF). The QToF-MS base peak chromatograms (BPC) of the TME and active DCMF in negative and positive modes are shown in Fig. 1. The corresponding LC-DAD chromatograms for both samples at 254, 280 and 330 nm are shown in Fig. 2. The ESI positive and negative ionization modes analysis using LC-QToF resulted in the tentative identification and characterization of 56 phytochemical compounds in *E. grantii* aerial parts in both samples with compounds **4**, **6**, and **12** present in low abundances in DCMF. The metabolites were tentatively described based on their UV spectra at 254, 280, and 330 nm, accurate mass spectra, and MS fragmentation patterns and/or by comparing the mass spectra of the metabolites to those recorded in the phytochemical dictionary of natural products database (CRC) and in published literature (Buckingham 2020). However, certain analytes cannot be easily ionized when soft ionization techniques are applied, leading to their low sensitivity using LC–MS analysis. Additionally, limited MS/MS fragmentation will usually hinder structural determination. It is challenging to effectively ionize neutral, low polarity, and non-polar compounds. Because of this low ionization nature with minimal or no UV activity, some of the isolated compounds were difficult to identify or confirm using LC-ESI-QToF method of analysis (Mitamura and Shimada 2001).

**Fig. 3:**
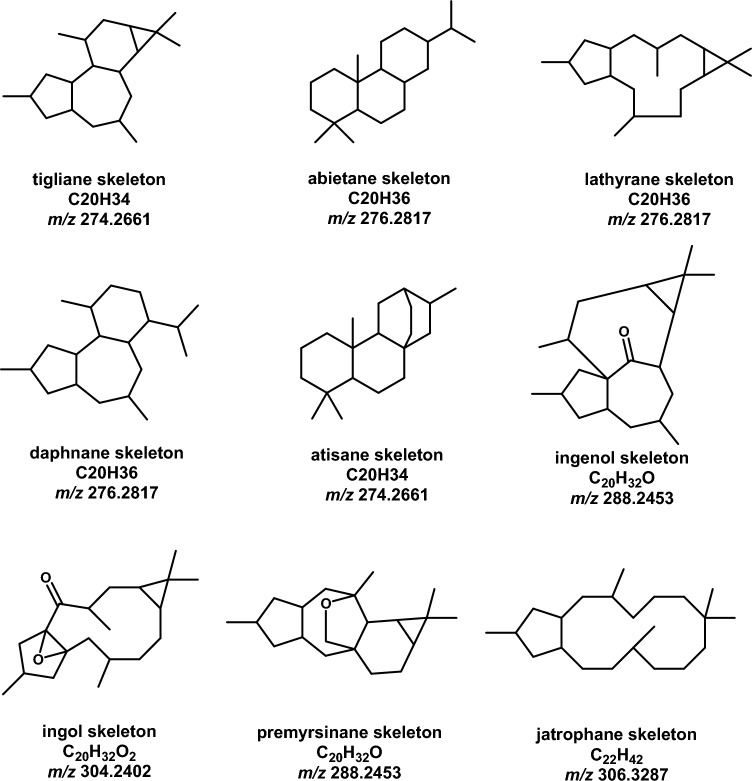
Skeletons of diterpenes and diterpenes esters identified in *E. grantii* extract

Most of the identified compounds are diterpenoids or diterpenoids esters with nine different skeletons: tigliane, ent-abietane, lathyrane (ingol), myrsinane, premyrsinane, ent-atisane, jatrophane, ingenol, and daphnane (Fig. 3). Two compounds, **8** and **11,** are bisnorsesquiterpenoids. Monoterpenoids like compound **16** (monoterpenoid lactone) and compound **7** (monoterpenoid iridoid) were also found, as well as some triterpenoids such as compounds **41** and **42**. Other secondary metabolites, including acids, lactams, coumarins, pyridine alkaloids and glycosides were identified (Table 3).

**Table 3:** Tentative identification and characterization of phytochemical compounds in DCMF and TME from *E. grantii* aerial parts using LC-QToF in positive and negative ionization modes

#	RT (min)	Compound Name ^[Refs.]^	CH_2_Cl_2_	MeOH	Mol. Formula	Exact mass [M]	Positive mode ion (adduct)	Fragment ions (+ve mode)	Negative mode ion (adduct)	Fragment ions (−ve mode)
1	3.51	Aminoshikimic acid(Mitamura and Shimada 2001; Buckingham 2020)	**ND**	**√**	C_7_H_11_NO_4_	173.0688	–	–	172.0618 (172.0615)* [M − H]^−^	–
2	3.65	Guvacine (Mitamura and Shimada 2001; Buckingham 2020)	**√**	**√**	C_6_H_9_NO_2_	127.0633	128.0705 (128.0706)* [M + H]^+^	–	–	–
3	3.83	4-Hydroxy-5-methoxy-3-methylene-2(1*H*)-pyrrolidinone (Mitamura and Shimada 2001; Buckingham 2020)	**√**	**√**	C_6_H_9_NO_3_	143.0582	144.0655 (144.0655) [M + H]^+^	103.0542, 84.0446, 77.0388		
4	4.73	Protocatechuic acid (Mitamura and Shimada 2001; Buckingham 2020)	**√**	**√**	C_7_H_6_O_4_	154.0266	–	–	153.0192 (153.0193) [M − H]^−^	109.0297 [M − H–CO_2_]^−^
5	5.0	Enamidin (Buckingham 2020)	**ND**	**√**	C_8_H_13_NO_4_	187.0845	–	–	186.0772 (186.0772) [M − H]^−^	–
6	5.14	Hydroxymethyl-pyrrolidine carboxylic acid (Buckingham 2020)	**√**	**√**	C_6_H_11_NO_3_	145.0739	146.0811 (146.0812) [M + H]^+^	118.0860 [M + H–CO]^+^, 114.0913 [M + H-O_2_]^+^	144.0667 (144.0666) [M − H]^−^	–
7	5.63	Loganetin (Buckingham 2020)	**ND**	**√**	C_11_H_16_O_5_	228.0998	–	–	227.0922 (227.0925) [M − H]^−^	183.1024 [M − H–CO_2_]^−^
8	5.72	Euphorbioside B (Buckingham 2020)	**ND**	**√**	C_19_H_34_O_9_	406.2203	429.2093 (429.2095) [M + Na]^+^	209.1535 [M + H–C_6_H_12_O_6_–H_2_O]^+^, 167.1066 [M + H–C_6_H_12_O_6_–H_2_O–C_3_H_6_]^+^	451.2189 (451.2185) [M + COOH]^−^	–
9	6.80	3-Hydroxy-7-methoxycoumarin (Buckingham 2020)	**ND**	**√**	C_10_H_8_O_4_	192.0423	–	–	191.0349 (191.0350) [M − H]^−^	–
10	7.3	3,4,5,19,20-pentahydroxy-1,6-ingenadien-9-one; (3β,4β,5β)-form, 19-*O*-β-D-glucopyranoside (Buckingham 2020)	**ND**	**√**	C_26_H_38_O_11_	526.2414	–	–	525.2337 (525.2341) [M − H]^−^	393.1552 [M − H–C_6_H_12_O_3_]^−^, 281.1393 [M − H–C_6_H_12_O_3_–C_5_H_4_O_3_]^−^
11	7.60	Euphorbioside A (Buckingham 2020)	**√**	**√**	C_19_H_32_O_9_	404.2046	422.2386 (422.2385) [M + NH_4_]^+^	227.1638 [M + H–C_6_H_10_O_6_]^+^, 209.1528 [M + H–C_6_H_10_O_6_–H_2_O]^+^, 183.1011 [M + H–C_6_H_10_O_6_–C_3_H_8_]^+^	449.2032 (449.2028) [M + COOH]^−^	215.1288 [M − H–C_8_H_12_O_5_]^−^, 203.0927 [M − H–C_10_H_16_O_4_]^−^, 157.0506 [C_7_H_8_O_4_–H]^−^, 113.0608 [C_7_H_8_O_4_–H–CO_2_]^−^
12	8.28	Phorbol (Buckingham 2020)	**√**	**√**	C_20_H_28_O_6_	364.1886	387.1779 (387.1778) [M + Na]^+^	347.1852 [M + H–H_2_O]^+^, 329.1744 [M + H–2H_2_O]^+^, 311.1641 [M + H–3H_2_O]^+^, 293.1536 [M + H–4H_2_O]^+^, 195.0805 [M + H-4H_2_O–C_6_H_10_O]^+^, 165.0694 [M + H–4H_2_O–C_6_H_10_O–CH_2_O]^+^	363.1810 (363.1813) [M − H]^−^	345.1708 [M − H–H_2_O]^−^, 315.1598 [M − H–H_2_O–CH_2_O]^−^
13	8.65	Euphelionolide A (Bicchi et al. 2001; Su et al. 2003)	**√**	**√**	C_20_H_28_O_5_	348.1937	–	–	393.1915 (393.1919) [M + COOH]^−^	-
14	8.81	Dehydroeuphoreppinol (Buckingham 2020)	**√**	**√**	C_20_H_30_O_7_	382.1992	–	–	427.1973 (427.1974) [M + COOH]^−^	-
15	10.0	Ingol (Buckingham 2020)	**√**	**√**	C_20_H_30_O_6_	366.2042	367.2120 (367.2115) [M + H]^+^	349.2013 [M + H–H_2_O]^+^, 331.1910 [M + H–2H_2_O]^+^, 313.1804 [M + H–3H_2_O]^+^, 285.1853 [M + H–3H_2_O–CO]^+^, 267.1738 [M + H–4H_2_O–CO]^+^, 197.1176 [M + H–2H_2_O–C_9_H_10_O]^+^	411.2032 (411.2034) [M + COOH]^−^	-
16	12.77		**√**	**√**						
17	10.26	Loliolide (Buckingham 2020)	**√**	**√**	C_11_H_16_O_3_	196.1099	197.1181 (197.1172) [M + H]^+^	179.1029 [M + H–H_2_O]^+^	–	–
18	11.44	Euphopiloside A (Buckingham 2020)	**ND**	**√**	C_26_H_38_O_10_	510.2465	533.2362 (533.2357) [M + Na]^+^	493.2430 [M + H–H_2_O]^+^, 475.2334 [M + H–2H_2_O]^+^, 449.2173 [M + H–2H_2_O–CH_2_]^+^, 391.2114 [M + H–2H_2_O–CH_2_–C_2_H_2_O_2_]^+^, 209.1528 [C_13_H_20_O_2_ + H]^+^	509.2387 (509.2392) [M − H]^−^	343.2124 [M − H–C_8_H_6_O_4_]^−^
19	11.83	Prostratin (Su et al. 2003)	**√**	**√**	C_22_H_30_O_6_	390.2042	413.1942 (413.1935) [M + Na]^+^	373.2015 [M + H–H_2_O]^+^, 355.1908 [M + H–2H_2_O]^+^, 313.1805 [M + H–2H_2_O–C_2_H_2_O]^+^, 295.1705 [M + H–3H_2_O–C_2_H_2_O]^+^	435.2025 (435.2024) [M + COOH]^−^	–
20	12.15	Trihydroxy-3-atisanone isomers (Buckingham 2020)	**√**	**√**	C_20_H_32_O_4_	336.2301	337.2366 (337.2373) [M + H]^+^	319.2265 [M + H–H_2_O]^+^, 301.2159 [M + H–2H_2_O]^+^, 283.2052 [M + H–3H_2_O]^+^, 249.1477 [M + H–C_5_H_12_O]^+^, 207.1374 [M + H–C_5_H_12_O–C_2_H_2_O]^+^, 193.1216 [M + H–C_5_H_12_O–C_2_H_2_O–CH_2_]^+^, 175.1113 [M + H–C_5_H_12_O–C_2_H_2_O–CH_2_–H_2_O]^+^, 133.1004 [M + H–C_5_H_12_O–2C_2_H_2_O–CH_2_–H_2_O]^+^	381.2271 (381.2283) [M + COOH]^−^	–
21	12.8		**√**	**√**						
22	12.54	Euphelionolide N/I/H (Buckingham 2020)	**√**	**√**	C_20_H_26_O_5_	346.1780	347.1850 (347.1853) [M + H]^+^	329.1743 [M + H–H_2_O]^+^, 315.1950 [M + H–H_2_O–O_2_]^+^, 267.1587 [M + H–C_5_H_4_O]^+^, 251.1629 [M + H–C_5_H_4_O–O]^+^, 207.1368 [M + H–C_5_H_4_O–O–C_2_H_4_O]^+^	391.1762 (391.1764) [M + COOH]^−^	289.1805 [M − H–H_2_O–C_2_O_2_]^−^
23	13.94		**√**	**√**						
24	13.30	17-Hydroxyingenol; 20-Deoxy, 3-*O*-β-D-glucopyranoside (Buckingham 2020)	**√**	**√**	C_26_H_38_O_10_	510.2465	–	–	555.2446 (555.2447) [M + COOH]^−^	367.2128 [M − H–C_6_H_6_O_4_]^−^, 359.1500 [M − H–C_6_H_14_O_4_]^−^, 315.1593 [M − H–C_6_H_14_O_4_–CO_2_]^−^, 255.1387 [M − H–C_6_H_14_O_4_–CO_2_–C_2_H_4_O_2_]^−^
25	14.45	Jolkinolide B (Buckingham 2020)	**√**	**√**	C_20_H_26_O_4_	330.1831	331.1903 (331.1904) [M + H]^+^	209.1302 [M + H–C_4_H_10_O_4_]^+^, 195.1149 [M + H–C_4_H_10_O_4_–CH_2_]^+^, 183.1154 [M + H–C_4_H_10_O_4_–C_2_H_2_]^+^, 169.0998 [M + H–C_4_H_10_O_4_–C_2_H_2_–CH_2_]^+^, 157.0997 [M + H–C_4_H_10_O_4_–2C_2_H_2_]^+^, 143.0840 [M + H–C_4_H_10_O_4_–2C_2_H_2_–CH_2_]^+^, 131.0842 [M + H–C_4_H_10_O_4_–3C_2_H_2_]^+^, 117.0688 [M + H–C_4_H_10_O_4_–3C_2_H_2_–CH_2_]^+^, 105.0689 [M + H–C_4_H_10_O_4_–4C_2_H_2_]^+^	375.1810 (375.1813) [M + COOH]^−^	–
26	16.0	Euphelionolide J/K (Buckingham 2020)	**√**	**√**	C_20_H_26_O_6_	362.1729	–	–	361.1659 (361.1657) [M − H]^−^	229.1234 [M − H–C_5_H_8_O_4_]^−^ 163.0764 [M − H–C_5_H_8_O_4_–C_5_H_6_]^−^, 123.0816 [M − H–C_5_H_8_O_4_–C_5_H_6_–C_2_O]^−^
27	17.54	4,15-Epoxy-3,7,8,12-tetrahydroxy-5-lathyren-14-one; (2β,3β,4β,5*E* ,7α,8α,12α,13α,15β)-form, 8-Tigloyl/ 7-angeloyl (Buckingham 2020)	**√**	**√**	C_25_H_36_O_7_	448.2461	–	–	493.2445 (493.2443) [M + COOH]^−^	337.2053 [M − H–C_6_H_6_O]^−^, 249.1163 [M − H–C_6_H_6_O–C_5_H_12_O]^−^
28	19.52	Decipinone C isomers (Buckingham 2020)	**√**	**√**	C_30_H_42_O_11_	578.2727	601.2623 (601.2619) [M + Na]^+^	561.2696 [M + H–H_2_O]^+^, 543.2602 [M + H–2H_2_O]^+^, 519.2590 [M + H–C_2_H_4_O_2_]^+^, 501.2481 [M + H–C_2_H_4_O_2_-H_2_O]^+^, 459.2377 [M + H–C_2_H_4_O_2_–H_2_O-C_2_H_2_O]^+^, 431.2088 [M + H–C_2_H_4_O_2_–H_2_O–C_2_H_2_O–C_2_H_4_]^+^, 413.1960 [M + H–C_2_H_4_O_2_–2H_2_O–C_2_H_2_O–C_2_H_4_]^+^, 371.1854 [M + H–C_2_H_4_O_2_–2H_2_O–2C_2_H_2_O–C_2_H_4_]^+^, 353.1750 [M + H–C_2_H_4_O_2_–3H_2_O–2C_2_H_2_O–C_2_H_4_]^+^, 311.1638 [M + H–C_2_H_4_O_2_–3H_2_O–3C_2_H_2_O–C_2_H_4_]^+^, 293.1535 [M + H–C_2_H_4_O_2_–4H_2_O–3C_2_H_2_O–C_2_H_4_]^+^, 265.1583 [M + H–C_2_H_4_O_2_–4H_2_O–3C_2_H_2_O–C_2_H_4_–CO]^+^, 223.1111 [M + H–C_2_H_4_O_2_–4H_2_O–4C_2_H_2_O–C_2_H_4_–CO]^+^, 195.1161 [M + H–C_2_H_4_O_2_–4H_2_O–4C_2_H_2_O–2C_2_H_4_–CO]^+^, 181.1007 [M + H–C_2_H_4_O_2_–4H_2_O–4C_2_H_2_O–2C_2_H_4_–CO–CH_2_]^+^	623.2704 (623.2709) [M + COOH]^−^	547.2548 [M − H–CH_2_O]^−^ 311.2228 [M − H–CH_2_O–C_9_H_18_O_6_]^−^, 309.2066 [M − H–CH_2_O–C_9_H_18_O_6_–H_2_]^−^
29	20.21		**√**	**√**						
30	21.07		**√**	**√**						
31	21.94		**√**	**√**						
32	20.65	4,15-Epoxy-3,7,8,12-tetrahydroxy-5-lathyren-14-one; (2β,3β,4β,5*E* ,7α,8α,12α,13α,15β)-form, Tetra-Ac isomers (Buckingham 2020)	**√**	**√**	C_28_H_38_O_10_	534.2465	557.2359 (557.2357) [M + Na]^+^	415.2119 [M + H–C_4_H_8_O_4_]^+^, 355.1904 [M + H–C_4_H_8_O_4_–C_2_H_4_O_2_]^+^, 295.1689 [M + H–C_4_H_8_O_4_–2C_2_H_4_O_2_]^+^, 207.1162 [M + H–C_4_H_8_O_4_–2C_2_H_4_O_2_–C_4_H_8_O_2_]^+^	–	–
33	22.1	Euphoppin A (Buckingham 2020)	**√**	**√**	C_31_H_44_O_11_	592.2884	593.2956 610.3220 (610.3222) [M + NH_4_]^+^ 615.2771 (615.2776) [M + Na]^+^	–	637.2868 (637.2866) [M + COOH]^−^	577.2682 [M − H–CH_2_]^−^, 545.2374 [M − H–CH_2_–CH_4_O]^−^, 381.1741 [M − H–CH_2_–CH_4_O–C_7_H_8_]^−^, 351.1636 [M − H–CH_2_–CH_4_O–C_7_H_8_–CH_2_O]^−^
34	22.1	4,15-Epoxy-3,7,8,12-tetrahydroxy-5-lathyren-14-one; (2β,3β,4β,5*E,* 7α,8α,12α,13α,15β)-form, 7/8/3-*O*-(Phenylacetyl), 3,8,12-tri-Ac (Buckingham 2020)	**√**	**√**	C_34_H_42_O_10_	610.2778	611.2852 (611.2851) [M + H]^+^ 628.3118 (628.3116) [M + NH_4_]^+^ 633.2667 (633.2670) [M + Na]^+^	551.2639 [M + H–C_2_H_4_O_2_]^+^, 509.2525 [M + H–C_2_H_4_O_2_–C_2_H_2_O]^+^, 491.2418 [M + H–C_2_H_4_O_2_–C_2_H_2_O–H_2_O]^+^, 449.2315 [M + H–C_2_H_4_O_2_–2C_2_H_2_O–H_2_O]^+^, 415.2105 [M + H–C_2_H_4_O_2_–C_2_H_2_O–H_2_O–C_6_H_4_]^+^, 373.2003 [M + H–C_2_H_4_O_2_–2C_2_H_2_O–H_2_O–C_6_H_4_]^+^, 313.1792 [M + H–2C_2_H_4_O_2_–2C_2_H_2_O–H_2_O–C_6_H_4_]^+^, 253.1577 [M + H–3C_2_H_4_O_2_–2C_2_H_2_O–H_2_O–C_6_H_4_]^+^, 161.0951 [M + H–3C_2_H_4_O_2_–2C_2_H_2_O–H_2_O–C_6_H_4_–C_7_H_8_]^+^	655.2753 (655.2760) [M + COOH]^−^	–
35	24.0		**√**	**√**						
36	25.4		**√**	**√**						
37	26.6		**√**	**√**						
38	22.47	Euphordraculoin K (Buckingham 2020)	**√**	**√**	C_34_H_44_O_11_	628.2884	651.2785 (651.2776) [M + Na]^+^	611.2861 [M + H–H_2_O]^+^, 569.2756 [M + H–H_2_O–C_2_H_2_O]^+^, 551.2646 [M + H–2H_2_O–C_2_H_2_O]^+^, 517.2809 [M + H–2H_2_O–C_2_H_2_O–CO]^+^, 433.2229 [M + H–2H_2_O–C_2_H_2_O–CO–C_7_H_8_O]^+^, 373.2018 [M + H–2H_2_O–C_2_H_2_O–CO–C_7_H_8_O–C_2_H_4_O_2_]^+^, 295.1701 [M + H–2H_2_O–C_2_H_2_O–CO–C_7_H_8_O–C_2_H_4_O_2_–C_2_H_6_O_3_]^+^	–	–
39	22.75	Euphorblin G (Buckingham 2020)	**√**	**√**	C_32_H_40_O_9_	568.2672	591.2580 (591.2565) [M + Na]^+^	549.2484 [M + H–H_2_–H_2_O]^+^	–	–
40	23.12	Euphorbiaproliferin C isomers (Buckingham 2020)	**√**	**√**	C_32_H_44_O_12_	620.2833	638.3178 (638.3171) [M + NH_4_]^+^ 643.2721 (643.2725) [M + Na]^+^	561.2697 [M + H–C_2_H_4_O_2_]^+^, 501.2486 [M + H–2C_2_H_4_O_2_]^+^, 473.2174 [M + H–2C_2_H_4_O_2_–C_2_H_4_]^+^, 413.1968 [M + H–3C_2_H_4_O_2_–C_2_H_4_]^+^, 353.1752 [M + H–4C_2_H_4_O_2_–C_2_H_4_]^+^, 325.1807 [M + H–4C_2_H_4_O_2_–C_2_H_4_-CO]^+^	665.2815 (665.2815) [M + COOH]^−^	559.2547 [M − H–C_2_H_4_O_2_]^−^
41	24.2		**√**	**√**						
42	23.32	4,15-Epoxy-3,7,8,12-tetrahydroxy-5-lathyren-14-one; (2β,3β,4β,5*E* ,7α,8α,12α,13α,15β)-form, 7/12/8-Tigloyl, 3,12-di-Ac/ 4,15-Epoxy-3,7,8,12-tetrahydroxy-5-lathyren-14-one; (2β,3β,4β,5*E,* 7α,8α,12α,13α,15β)-form, 7-Angeloyl, 3,12-di-Ac (Buckingham 2020)	**√**	**√**	C_29_H_40_O_9_	532.2672	555.2566 (555.2565) [M + Na]^+^	515.2650 [M + H–H_2_O]^+^, 473.2539 [M + H–H_2_O–C_2_H_2_O]^+^, 373.2014 [M + H–H_2_O–C_2_H_2_O–C_5_H_8_O_2_]^+^, 313.1804 [M + H–H_2_O–C_2_H_2_O–C_5_H_8_O_2_–C_2_H_4_O_2_]^+^	–	–
43	23.71	Euphorbiaproliferin D (Buckingham 2020)	**√**	**√**	C_36_H_44_O_12_	668.2833	691.2727 (691.2725) [M + Na] +	651.2792 [M + H–H_2_O]^+^, 609.2704 [M + H–H_2_O–C_2_H_2_O]^+^, 549.2485 [M + H–H_2_O–C_2_H_2_O–C_2_H_4_O_2_]^+^, 489.2267 [M + H–H_2_O–C_2_H_2_O–2C_2_H_4_O_2_]^+^, 473.2174 [M + H–H_2_O–C_2_H_2_O–C_2_H_4_O_2_–C_6_H_4_]^+^, 413.1956 [M + H–H_2_O–C_2_H_2_O–2C_2_H_4_O_2_–C_6_H_4_]^+^, 385.2005 [M + H–H_2_O–C_2_H_2_O–2C_2_H_4_O_2_–C_6_H_4_-CO]^+^, 353.1740 [M + H–H_2_O–C_2_H_2_O–2C_2_H_4_O_2_–C_6_H_4_–CO–CH_4_O]^+^, 325.1789 [M + H–H_2_O–C_2_H_2_O–2C_2_H_4_O_2_–C_6_H_4_–2CO–CH_4_O]^+^, 311.1632 [M + H–H_2_O–C_2_H_2_O–2C_2_H_4_O_2_–C_6_H_4_–2CO–CH_4_O–CH_2_]^+^, 293.1527 [M + H–2H_2_O–C_2_H_2_O–2C_2_H_4_O_2_–C_6_H_4_–2CO–CH_4_O–CH_2_]^+^, 275.1421 [M + H–3H_2_O–C_2_H_2_O–2C_2_H_4_O_2_–C_6_H_4_–2CO–CH_4_O–CH_2_]^+^, 265.1575 [M + H–2H_2_O–C_2_H_2_O–2C_2_H_4_O_2_–C_6_H_4_-3CO–CH_4_O–CH_2_]^+^, 247.1469 [M + H–3H_2_O–C_2_H_2_O–2C_2_H_4_O_2_–C_6_H_4_–3CO–CH_4_O–CH_2_]^+^, 223.1105 [C_16_H_14_O + H]^+^, 195.1154–[C_16_H_14_O–CO + H]^+^, 181.1000 [C_16_H_14_O–CO–CH_2_ + H]^+^	713.2828 (713.2817) [M + COOH]^−^	623.2489 [M − H–C_2_H_4_O]^−^, 469.2567 [M − H–C_8_H_6_O_6_]^−^, 367.1945 [C_16_H_32_O_9_–H]^−^, 341.1760 [C_16_H_32_O_9_–H–C_2_H_2_]^−^, 311.1684 [C_16_H_32_O_9_–H–C_2_H_2_–CH_2_O]^−^
44	24.52									
45	25.0	Euphorblin A/H/J (Mitamura and Shimada 2001)	**√**	**√**	C_34_H_42_O_11_	626.2727	649.2617 (649.2619) [M + Na]^+^	593.2747 [M + H–H_2_O_2_]^+^, 567.2591 [M + H–H_2_O_2_–C_2_H_2_]^+^, 551.2650 [M + H–H_2_O_2_–C_2_H_2_O]^+^, 533.2551 [M + H–H_2_O_2_–C_2_H_2_O–H_2_O]^+^, 507.2379 [M + H–H_2_O_2_–C_2_H_2_O–H_2_O–C_2_H_2_]^+^, 491.2430 [M + H–H_2_O_2_–2C_2_H_2_O–H_2_O]^+^, 415.2117 [M + H–H_2_O_2_–2C_2_H_2_O–H_2_O–C_6_H_14_]^+^, 355.1905 [M + H–H_2_O_2_–2C_2_H_2_O–H_2_O–C_6_H_14_–C_2_H_4_O_2_]^+^, 337.1795 [M + H–H_2_O_2_–2C_2_H_2_O–2H_2_O–C_6_H_14_–C_2_H_4_O_2_]^+^, 295.1694 [M + H–H_2_O_2_–3C_2_H_2_O–2H_2_O–C_6_H_14_–C_2_H_4_O_2_]^+^, 249.1634 [M + H–H_2_O_2_–3C_2_H_2_O–2H_2_O–C_6_H_14_–C_2_H_4_O_2_–C_2_H_2_O]^+^	671.2701 (671.2709) [M + COOH]^−^	–
46	26.8	Euphoreppine A (Buckingham 2020)	**√**	**√**	C_35_H_48_O_13_	676.3095	699.2993 (699.2987) [M + Na]^+^	557.2745 [M + H–C_4_H_8_O_4_]^+^, 503.2640 [M + H–C_4_H_8_O_4_–C_3_H_2_O]^+^, 457.2221 [M + H–C_4_H_8_O_4_–C_3_H_2_O–C_2_H_6_O]^+^, 415.2113 [M + H–C_4_H_8_O_4_–C_3_H_2_O–C_2_H_6_O–C_2_H_2_O]^+^, 397.2010 [M + H–C_4_H_8_O_4_–C_3_H_2_O–C_2_H_6_O–C_2_H_2_O–H_2_O]^+^, 355.1899 [M + H–C_4_H_8_O_4_–C_3_H_2_O–C_2_H_6_O–2C_2_H_2_O–H_2_O]^+^, 337.1792 [M + H–C_4_H_8_O_4_–C_3_H_2_O–C_2_H_6_O–2C_2_H_2_O–2H_2_O]^+^, 295.2264 [C_18_H_30_O_3_ + H]^+^, 277.2156 [C_18_H_30_O_3_ + H–H_2_O]^+^, 249.1628 [C_18_H_30_O_3_ + H–H_2_O–CO]^+^	–	–
47	27.05	4,15-Epoxy-3,7,8,12-tetrahydroxy-5-lathyren-14-one; (2β,3β,4β,5*E,* 7α,8α,12α,13α,15β)-form, 8-*O*-(2-Methylbutanoyl), 3,7,12-tri-Ac isomers (Buckingham 2020)	**√**	**√**	C_31_H_44_O_10_	576.2934	599.2827 (599.2827) [M + Na]^+^	517.2797 [M + H–C_2_H_4_O_2_]^+^, 431.2214 [M + H–C_2_H_4_O_2_–CH_10_O_4_]^+^, 415.2113 [M + H–C_2_H_4_O_2_–C_5_H_10_O_2_]^+^, 373.2004 [M + H–C_2_H_4_O_2_–C_5_H_10_O_2_–C_2_H_2_O]^+^, 355.1898 [M + H–C_2_H_4_O_2_–C_5_H_10_O_2_–C_2_H_2_O–H_2_O]^+^, 313.1795 [M + H–C_2_H_4_O_2_–C_5_H_10_O_2_–2C_2_H_2_O–H_2_O]^+^, 295.1691 [M + H–C_2_H_4_O_2_–C_5_H_10_O_2_–2C_2_H_2_O–2H_2_O]^+^, 279.1587 [M + H–C_2_H_4_O_2_–C_5_H_10_O_2_–C_2_H_2_O–H_2_O–C_6_H_4_]^+^, 267.1733 [M + H–C_2_H_4_O_2_–C_5_H_10_O_2_–2C_2_H_2_O–2H_2_O–CO]^+^, 163.0742 [C_10_H_10_O_2_ + H]^+^	–	–
48	27.72		**√**	**√**						
49	27.2	Synagrantol A (Buckingham 2020)	**√**	**√**	C_36_H_44_O_11_	652.2884	670.3224 (670.3222) [M + NH_**4**_**]**^**+**^ 675.2775 (675.2776) [M + Na]^+^	593.2742 [M + H–C_2_H_4_O_2_]^+^, 575.2640 [M + H–C_2_H_4_O_2_–H_2_O]^+^, 533.2525 [M + H–C_2_H_4_O_2_–H_2_O–C_2_H_2_O]^+^, 491.2424 [M + H–C_2_H_4_O_2_–H_2_O–2C_2_H_2_O]^+^, 473.2321 [M + H–C_2_H_4_O_2_–2H_2_O–2C_2_H_2_O]^+^, 431.2210 [M + H–C_2_H_4_O_2_–2H_2_O–3C_2_H_2_O]^+^, 355.1899 [M + H–C_2_H_4_O_2_–2H_2_O–3C_2_H_2_O–C_6_H_4_]^+^, 337.1795 [M + H–C_2_H_4_O_2_–3H_2_O–3C_2_H_2_O–C_6_H_4_]^+^, 313.1794 [M + H–C_2_H_4_O_2_–2H_2_O–4C_2_H_2_O–C_6_H_4_]^+^, 295.1690 [M + H–C_2_H_4_O_2_–3H_2_O–4C_2_H_2_O–C_6_H_4_]^+^, 277.1591 [M + H–C_2_H_4_O_2_–4H_2_O–4C_2_H_2_O–C_6_H_4_]^+^, 267.1736 [M + H–C_2_H_4_O_2_–3H_2_O–4C_2_H_2_O–C_6_H_4_–CO]^+^, 259.1777 [M + H–C_2_H_4_O_2_–5H_2_O–4C_2_H_2_O–C_6_H_4_]^+^, 249.1634 [M + H–C_2_H_4_O_2_–4H_2_O–4C_2_H_2_O–C_6_H_4_–CO]^+^	–	–
50	28.15		**√**	**√**						
51	27.42	Serrulatin A (Buckingham 2020)	**√**	**√**	C_38_H_48_O_12_	696.3146	719.3042 (719.3038) [M + Na]^+^	637.3015 [M + H–C_2_H_4_O_2_]^+^, 559.2908 [M + H–C_7_H_6_O_3_]^+^, 499.2690 [M + H–C_7_H_6_O_3_–C_2_H_4_O_2_]^+^, 457.2224 [M + H–C_7_H_6_O_3_–C_2_H_4_O_2_–C_3_H_6_]^+^, 397.2016 [M + H–C_7_H_6_O_3_–2C_2_H_4_O_2_–C_3_H_6_]^+^, 355.1902 [M + H–C_7_H_6_O_3_–2C_2_H_4_O_2_–C_3_H_6_–C_2_H_2_O]^+^, 337.1799 [M + H–C_7_H_6_O_3_–2C_2_H_4_O_2_–C_3_H_6_–C_2_H_2_O–H_2_O]^+^, 295.1694 [M + H–C_7_H_6_O_3_–2C_2_H_4_O_2_–C_3_H_6_–2C_2_H_2_O–H_2_O]^+^, 277.1587 [M + H–C_7_H_6_O_3_–2C_2_H_4_O_2_–C_3_H_6_–2C_2_H_2_O–2H_2_O]^+^, 181.1215 [C_11_H_16_O_2_ + H]^+^	–	–
52	28.0	Synagrantol B (Buckingham 2020)	**√**	**√**	C_30_H_40_O_7_	512.2774	530.3117 (530.3112) [M + NH_4_]^+^ 535.2669 (535.2666) [M + Na]^+^	–	–	–
53	28.8	4,15-Epoxy-3,7,8,12-tetrahydroxy-5-lathyren-14-one; (2β,3β,4β,5*E,* 7α,8α,12α,13α,15β)-form, 3-Benzoyl, 8-tigloyl, 12-Ac/4,15-Epoxy-3,7,8,12-tetrahydroxy-5-lathyren-14-one; (2β,3β,4β,5*E* ,7α,8α,12α,13α,15β)-form, 8-Benzoyl, 3-tigloyl, 12-Ac/ 4,15-Epoxy-3,7,8,12-tetrahydroxy-5-lathyren-14-one; (2α,3β,4β,5*E* ,7α,8α,12α,13α,15β)-form, 3-Benzoyl, 8-tigloyl, 12-Ac (Buckingham 2020)	**√**	**√**	C_34_H_42_O_9_	594.2829	617.2730 (617.2721) [M + Na]^+^	535.2682 [M + H–C_2_H_4_O_2_]^+^, 475.2471 [M + H–2C_2_H_4_O_2_]^+^, 357.2050 [M + H–2C_2_H_4_O_2_–C_8_H_6_O]^+^, 339.1946 [M + H–2C_2_H_4_O_2_–C_8_H_6_O–H_2_O]^+^, 297.1841 [M + H–2C_2_H_4_O_2_–C_8_H_6_O–H_2_O–C_2_H_2_O]^+^, 279.1731 [M + H–2C_2_H_4_O_2_–C_8_H_6_O–H_2_O–C_2_H_2_O–H_2_O]^+^, 269.1886 [M + H–2C_2_H_4_O_2_–C_8_H_6_O–H_2_O–C_2_H_2_O–CO]^+^, 251.1781 [M + H–2C_2_H_4_O_2_–C_8_H_6_O–H_2_O–C_2_H_2_O–CO–H_2_O]^+^, 239.1415 [M + H–2C_2_H_4_O_2_–C_8_H_6_O–H_2_O–C_2_H_2_O–CO–C_2_H_6_]^+^, 209.1308 [M + H–2C_2_H_4_O_2_–C_8_H_6_O–H_2_O–C_2_H_2_O–CO–C_2_H_6_–CH_2_O]^+^	–	–
54	31.1	Friedelinol (Buckingham 2020)	**√**	**√**	C_30_H_52_O	428.4018	429.4110 (429.4091) [M + H]^+^	–	–	–
55	34.7	Friedelin/ Cycloartenol/Euphol (Buckingham 2020)	**√**	**√**	C_30_H_50_O	426.3862	–	–	425.3755 (424.3789) [M − H]^−^	–
56	35.05									

*Theoretical accurate mass; Compound #**4, 6, 12** are present in low abundances in dichloromethane fraction (DCMF)

### In vivo anti-inflammatory activity of the active DCMF

#### Biochemical study

For seven days, free pathogenic male albino rats were given DCMF (200 and 300 mg/kg b.wt., oral), as well as dexamethasone positive control (5 mg/kg, i.p.). On the 7^th^ day, 1 h after the last treatments, LPS (10 mg/kg b.wt., i.p.) was given to induce ALI. The levels of inflammatory cytokines were measured as key markers to judge the degree of systemic inflammatory responses. The results showed that the LPS-treated group had significant higher levels (*P* < 0.001) of pro-inflammatory cytokines (TNF-α, IL-6, IL-1β, and MPO) compared to the control group (Table 4). These results demonstrated that regular doses of DCMF (200 and 300 mg/kg) combined with LPS had a potent anti-inflammatory effect, as evidenced by the decreased levels of pro-inflammatory cytokines compared to the control and dexamethasone-treated groups.

**Table 4:** Pulmonary pro-inflammatory cytokines examination (TNF-α, IL-6, IL-β1 and MPO)

Group	TNF-α	IL-6	IL-β1	MPO
Control	34.07 ± 1.157	26.13 ± 0.554	28.47 ± 1.105	23.47 ± 0.6360
LPS (10 mg\kg)	157.0 ± 4.917^***a^	126.1 ± 3.75^***a^	112.2 ± 3.143^***a^	102.4 ± 3.496^***a^
Dexamethasone (5 mg\kg) + LPS	91.27 ± 1.940^***b^	58.87 ± 1.38^***b^	48.77 ± 1.995^***b^	45.50 ± 2.926^***b^
DCMF 200 mg/kg + LPS	119.2 ± 3.231^***b^	91.63 ± 3.94^***b^	81.13 ± 2.677^***b^	73.57 ± 2.685^***b^
DCMF 300 mg/kg + LPS	81.83 ± 1.994^***b^	53.13 ± 2.00^***b^	41.73 ± 1.877^***b^	41.33 ± 1.041^***b^
DCMF 200 mg/kg	128.7 ± 2.055	70.93 ± 2.34	62.47 ± 2.829	37.93 ± 2.010
DCMF 300 mg/kg	92.73 ± 2.083	61.33 ± 3.15	53.63 ± 3.072	28.60 ± 0.9866

Data are presented as mean value ± standard error of mean (SEM) of 5 rats. ^*^P (< 0.05) significant, ^**^P (< 0.01) highly significant, ^***^P (< 0.001) very highly significant

The activities of antioxidant enzymes (SOD, CAT, and GSH activities) were presented in Table 5**,** in addition to the data of non-oxidant enzyme (MDA). There was a significant difference (*P* < 0.05) between the LPS-treated, control, and dexamethasone-treated groups. The combination of DCMF (300 mg/kg) and the LPS-treated group resulted in highly significant increases in SOD, CAT activities, and GSH and a negative highly significant increase in MDA (*P* < 0.001).

**Table 5:** Pulmonary antioxidant and non-oxidant examination

Group	SOD	Catalase	GSH	MDA
Control	0.8450 ± 0.009	0.3610 ± 0.003	0.6110 ± 0.064	18.95 ± 1.84
LPS (10 mg\kg)	0.4713 ± 0.041^*a^	0.2153 ± 0.001^*a^	0.4737 ± 0.038^*a^	42.15 ± 0.800^***a^
Dexamethasone (5 mg/kg) + LPS	0.8047 ± 0.041^*b^	0.098 ± 0.006^*b^	1.174 ± 0.1565^**b^	20.69 ± 2.49^***b^
DCMF 200 mg/kg + LPS	0.8623 ± 0.052^**b^	0.1990 ± 0.024^*b^	0.4890 ± 0.083^**b^	27.05 ± 1.90^***b^
DCMF 300 mg/kg + LPS	1.052 ± 0.109^***b^	0.4507 ± 0.030^***b^	0.9930 ± 0.182^**b^	16.15 ± 2.66^***b^
DCMF 200 mg/kg	0.6437 ± 0.049	0.3170 ± 0.008	0.4517 ± 0.104	22.89 ± 1.15
DCMF 300 mg/kg	0.5747 ± 0.075	0.4157 ± 0.032	0.1960 ± 0.030	31.15 ± 0.808

Data are presented as mean value ± standard error of mean (SEM) of 5 rats. ^*^P (< 0.05) significant, ^**^P (< 0.01) highly significant, ^***^P (< 0.001) very highly significant

In Table 6, real-time PCR data for P38MAPK and CY450E2 genes expression in lung tissues revealed a highly significant difference when comparing the LPS-treated group with the control group (*P* < 0.001). The difference was also highly significant (*P* < 0.001) when comparing all combinations of (LPS + dexamethasone, LPS + DCMF, 200 mg/kg, and LPS + DCMF, 300 mg/kg).

**Table 6:** Pulmonary molecular identification of P38 MAPK and CY450P2E1 genes

Groups	P38 MAPK	CY450P2E1
	Fold change	
Control	–	–
LPS (10 mg/kg)	9.682 ± 0.1741^***a^	12.68 ± 0.2038^***a^
Dexamethasone (5 mg/kg) + LPS	3.585 ± 0.2314^***b^	5.345 ± 0.3346^***b^
DCMF 200 mg /kg + LPS	4.920 ± 0.06097^***b^	5.699 ± 0.1732^***b^
DCMF 300 mg/kg + LPS	2.587 ± 0.2115^***b^	3.569 ± 0.3478^***b^
DCMF 200 mg/kg	0.7900 ± 0.005774	0.8896 ± 0.01733
DCMF 300 kg/kg	0.6343 ± 0.02793	0.7657 ± 0.02944

Data are presented as mean value ± standard error of mean (SEM) of 5 rats. ^*^P (< 0.05) significant, ^**^P (< 0.01) highly significant, ^***^P (< 0.001) very highly significant

**Fig. 4:**
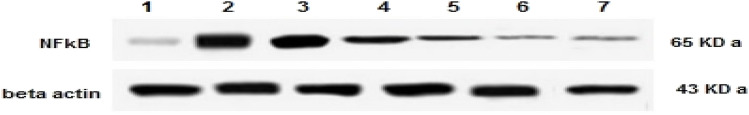
Western blot of Nuclear Factor-kappa B (NF-κB) in lung. 1 Control, 2 LPS (10 mg\kg), 3 Dexa (5 mg\kg) + LPS, 4 (DCMF-200 mg\kg) + LPS, and 5 (DCMF-300 mg\kg) + LPS, 6 (DCMF200mg\kg) + LPS, and 7 (DCMF300mg\kg) + LPS. Quantitation after normalization with Beta-actin (A.U.)

Moreover, the western blotting of lung tissue, represented in Table 7 and Fig. 4, showed a high degree of significance in the LPS-treated group compared to the control group. The results showed that the combined groups had significantly improved in comparison to the LPS-treated group.

**Table 7:** Western blot of pulmonary nuclear factor-kappa B (NF-κB)

Group	Nf-ĸB
Control	0.970 ± 0.005
LPS (10 mg/kg)	3.973 ± 0.014^***a^
Dexamethasone (5 mg/kg) + LPS	3.907 ± 0.048
DCMF (200 mg/kg) + LPS	2.003 ± 0.057^***b^
DCMF (300 mg/kg) + LPS	1.533 ± 0.242^***b^
DCMF (200 mg/kg)	0.976 ± 0.008
DCMF (300 mg/kg)	0.950 ± 0.0057

The ratio of the arbitrary units (AU) of the internal control to those of the antigen was used to calculate the numerical values of the relative band intensities

Data are presented as mean value ± standard error of mean (SEM) of 5 rats. ^*^P (< 0.05) significant, ^**^P (< 0.01) highly significant, ^***^P (< 0.001) very highly significant

#### Histopathological Examination

##### H&E results

**Fig. 5:**
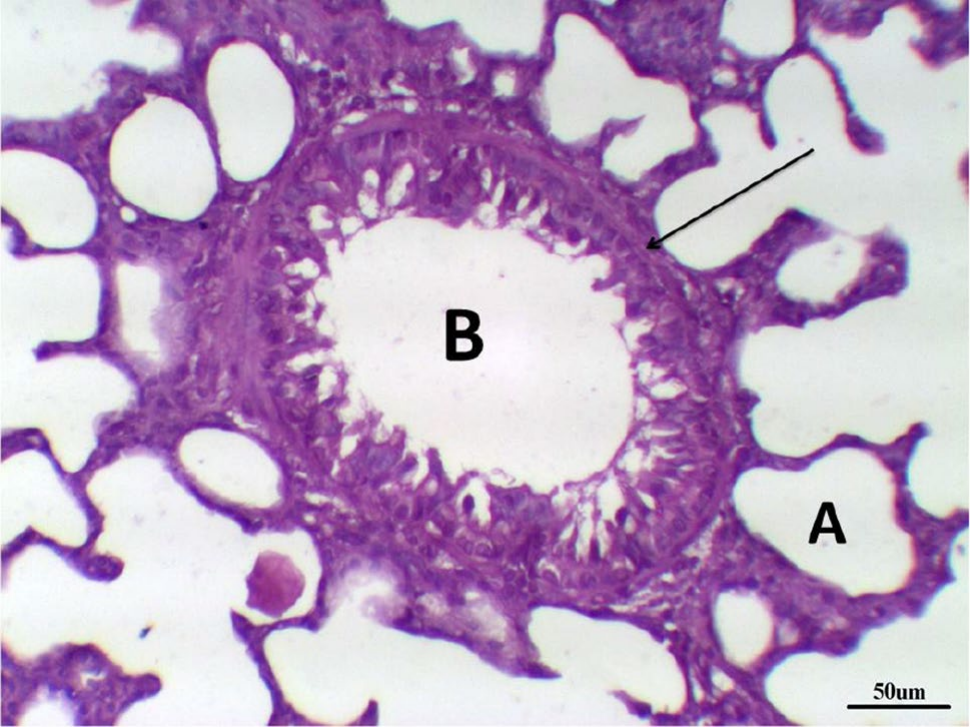
Lung control group stained with H & E revealed: Normal tissue architecture, The terminal bronchioles were lined by wavy ciliated simple columnar with underlying lamina propria (**B**), continuous musculosa layer (arrow) and thin adventitia, thin-walled air-filled alveoli and normal alveolar sacs (**A**), ×200

Histological examination of lung tissue in the control group revealed normal tissue architecture. The alveoli were thin-walled, air-filled and had normal alveolar sacs. The thin inter-alveolar septa had numerous capillaries and minimal connective tissue. The terminal bronchioles were lined by wavy ciliated simple columnar or cuboidal epithelium with underlying lamina propria, continuous musculosa layer, and thin adventitia. The pulmonary blood vessels were also normal (Fig. 5 and Table 8).

**Table 8:** Histological scoring of pulmonary inflammation

Groups	Incidence of congestion	Incidence of hemorrhage	Severity of neutrophil infiltration	Proportion of airspace area
Control	1/8	0/8	–	9.22%
LPS (10 mg/kg)	5/8	2/8	++++	2.66%
dexamethasone (5 mg/kg) + LPS	4/8	1/8	++	4.89%
DCMF 200 mg/kg + LPS	3/8	1/8	++	7.24%
DCMF 300 mg/kg + LPS	1/8	0/8	+	9.12%
DCMF 200 mg/kg	1/8	0/8	+	10.47%
DCMF 300 mg/kg	2/8	0/8	–	10.67%

The same position of the lungs in eight mice was observed through a microscope, and the histological lesion of the lung was evaluated through the incidence of congestion, inflammation and hemorrhage, severity scoring of neutrophil infiltrate, and proportion of airspace areas. The level of severity was judged from − to +  +  +  + , which represented none to severe. The airspace proportion was the ratio of the airspace area and the total area of one view under 400× magnifications (Wang et al. 2018)

**Fig. 6:**
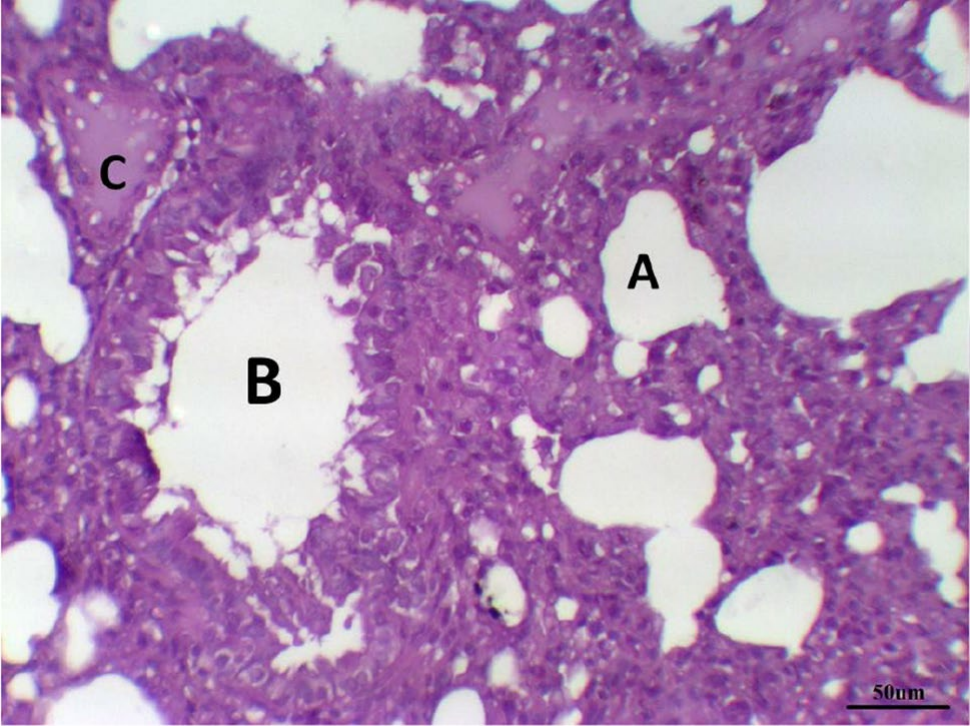
LPS group stained with H & E revealed: Thickened intra- alveolar septum with congestion, edema, lymphocytic infiltration (**A**), Terminal bronchioles were lined with atrophied vacuolated cuboidal epithelium (**B**) infiltrated with multiple inflammatory, Dilated congested blood vessel (**C**), ×200

The histological examination of lung tissues in the LPS group revealed collapsed alveoli in some areas, while other areas showed emphysema. Thickened intra- alveolar septum was noticed widespread with congestion, edema, lymphocytic infiltration, and eosinophilic material, especially at the peripheral lung tissue. Distorted alveolar architecture was detected. Terminal bronchioles were lined with atrophied vacuolated cuboidal epithelium; meanwhile, many areas showed desquamated epithelium with distorted architecture. The walls of the bronchioles were infiltrated with multiple inflammatory cells that interrupted the musculosa and invaded the mucosa. Mild hypertrophy of smooth muscles and thickened adventitia were demonstrated. Moderate to severe Peri-bronchial edema and inflammatory cells mainly neutrophils, lymphocytes, sometimes giant cells and basophils, were noticed. Severely thickened dilated congested pulmonary blood vessels were illustrated with intra-vascular edema, congestion. Peri-vascular edema and inflammatory cells were seen mainly neutrophils with distorted architecture (Fig. 6) and Table 8.

**Fig. 7:**
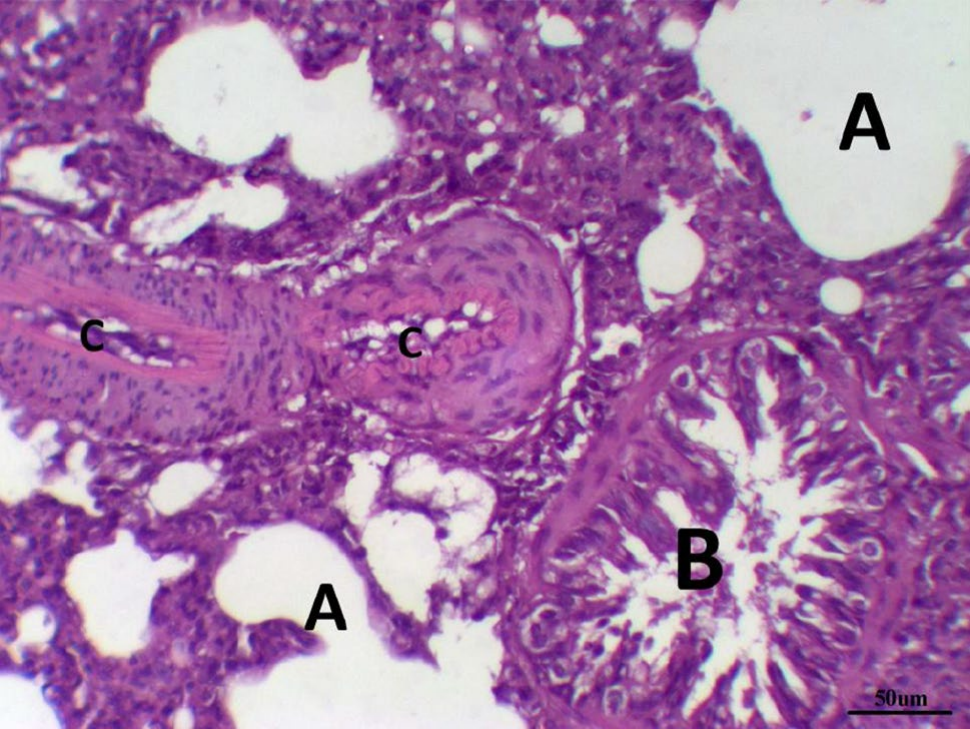
Lung LPS group treated with dexamethasone stained with H & E revealed: Emphysematous alveoli, thickened intra- alveolar septum (**A**), desquamated epithelium with distorted architecture of the bronchioles (**B**), hypertrophied dilated congested pulmonary blood vessels (**C**), ×200

The histological examination of lung tissues in the LPS group treated with dexamethasone revealed collapsed alveoli in some areas, while other areas showed emphysema. Thickened intra- alveolar septum was noticed widespread with congestion, edema, and lymphocytic infiltration. Distorted alveolar architecture was detected. The terminal bronchioles were lined with atrophied vacuolated cuboidal epithelium; meanwhile, many bronchioles showed desquamated epithelium with distorted architecture. The walls of the bronchioles were infiltrated with multiple inflammatory cells that interrupted the musculosa and invaded the mucosa. Mild hypertrophy of smooth muscles and thickened adventitia were demonstrated. Moderate to severe Peri-bronchial edema and inflammatory cells, mainly neutrophils, lymphocytes, sometimes giant cells, and basophils, were noticed. Severely hypertrophied dilated congested pulmonary blood vessels were illustrated with intra-vascular edema and congestion. Peri-vascular edema and inflammatory cells were seen mainly neutrophils with distorted architecture (Fig. 7 and Table 8).

**Fig. 8:**
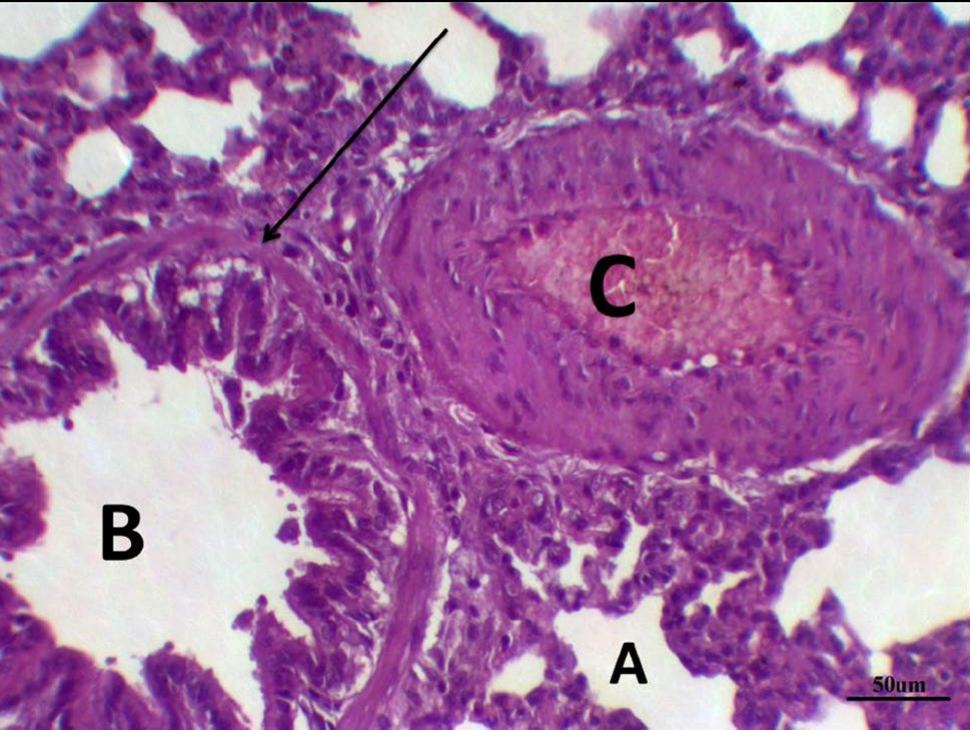
Lung LPS group treated with DCMF 200 mg/kg stained with H & E revealed: Collapsed alveoli (**A**), with thickened intra- alveolar septum and distorted alveolar architecture. Terminal bronchioles were lined with wavy ciliated cuboidal epithelium (**B**), musculosa layer (arrow). Moderate dilated hypertrophied congested blood vessels (**C**), ×200

The histological examination of lung tissues in the LPS group treated with DCMF, 200 mg/kg, revealed collapsed alveoli in some areas, while other areas showed emphysema. Thickened intra- alveolar septum was noticed in many areas, and distorted alveolar architecture was detected in many areas. The terminal bronchioles were lined with wavy ciliated cuboidal epithelium; meanwhile, and bronchioles showed desquamated epithelium with sub-epithelial edema. Moderate peri-bronchial inflammatory cells were noticed. Moderate dilated hypertrophied congested pulmonary blood vessels were illustrated with intra-vascular edema and congestion. Peri-vascular edema and inflammatory cells were seen mainly neutrophils with distorted architecture (Fig. 8 and Table 8).

**Fig. 9:**
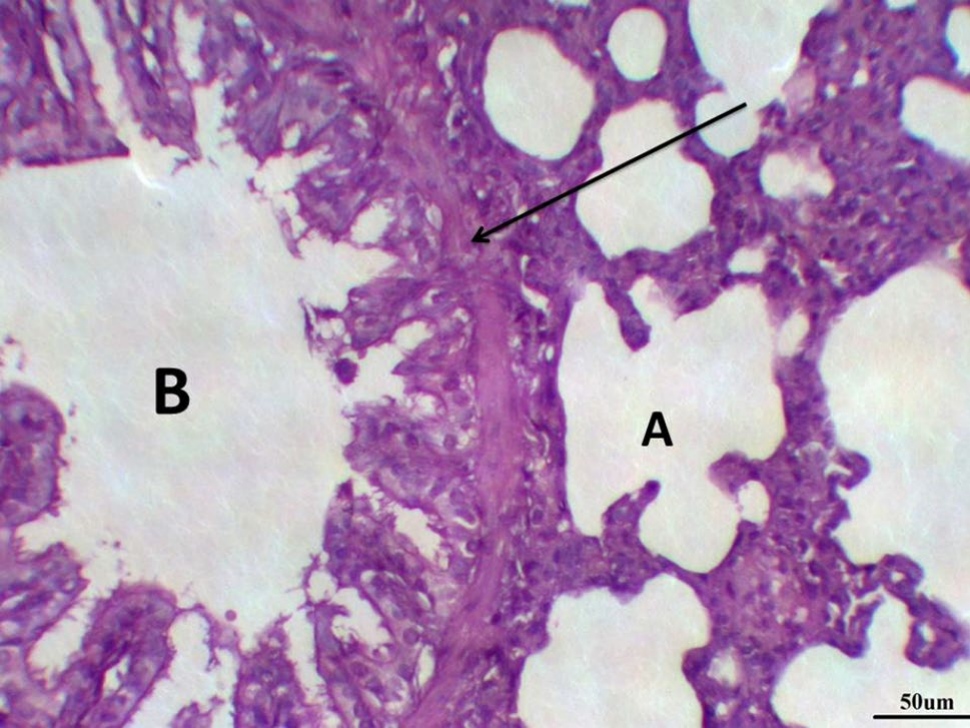
lung LPS group treated with DCMF 300 mg/kg stained with H & E revealed: thin-walled air-filled alveoli (**A**), the terminal bronchioles were lined by wavy ciliated simple cuboidal epithelium (**B**) continuous musculosa layer (arrow) and thin adventitia, ×200

The histological examination of lung tissues treated with DCMF, 200 mg/kg, revealed normal tissue architecture. The alveoli were thin-walled, air-filled, and had normal alveolar sacs. The thin inter-alveolar septum was illustrated, with peripheral lung zones thickened inter-alveolar septum. The terminal bronchioles were lined by wavy ciliated simple cuboidal epithelium with underlying lamina propria, continuous musculosa layer, and thin adventitia. Pulmonary blood vessels were mildly dilated hypertrophied (Fig. 9 and Table 8).

**Fig. 10:**
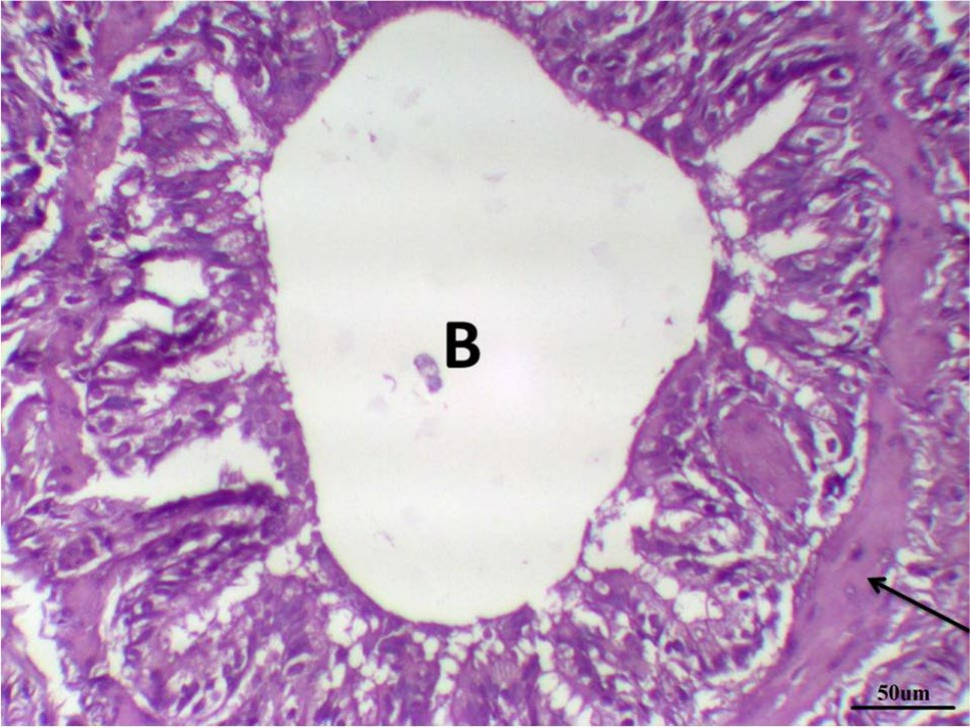
Lung treated with DCMF 200 mg/kg stained with H & E revealed: The terminal bronchioles (**B**) were lined by wavy ciliated simple columnar with underlying lamina propria, continuous musculosa layer (arrow) and thin adventitia, ×200

The lung treated with DCMF, 200 mg/kg revealed normal tissue architecture, which was demonstrated by thin-walled air-filled alveoli and normal alveolar sacs. The thin inter-alveolar septa had numerous capillaries and minimal connective tissue. The terminal bronchioles were lined by wavy ciliated simple columnar or cuboidal epithelium with underlying lamina propria, continuous musculosa layer and thin adventitia. Pulmonary blood vessels were moderately dilated (Fig. 10 and Table 8).

**Fig. 11:**
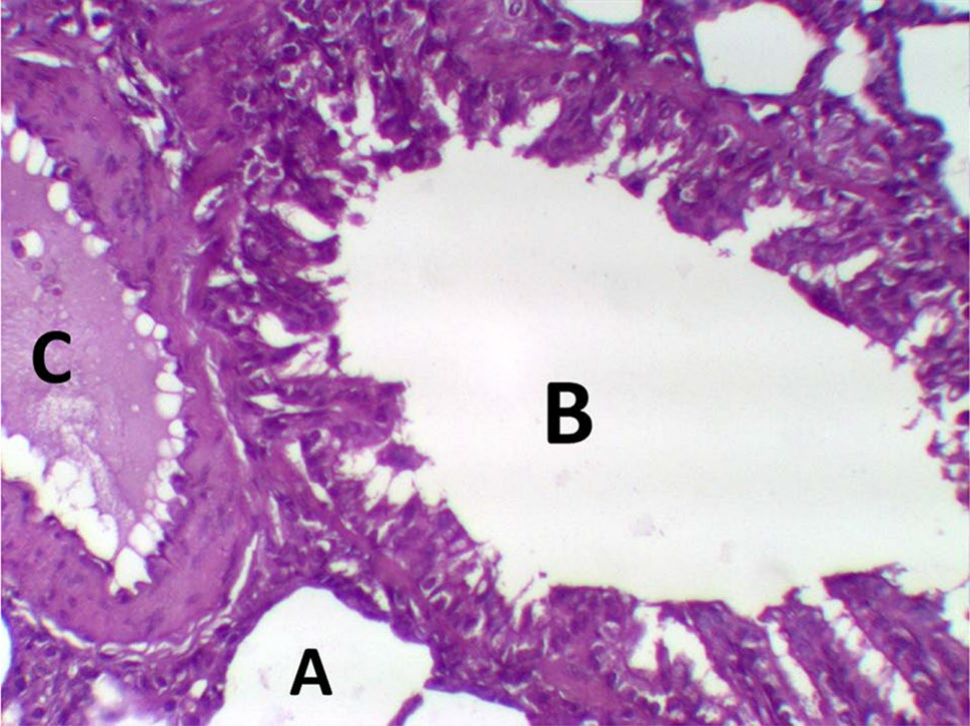
lung treated with DCMF 300 mg/kg stained with H & E revealed: The terminal bronchioles were lined by wavy ciliated cuboidal epithelium with underlying lamina propria (**B**), thin-walled air-filled alveoli (**A**), blood vessels were mildly dilated (C), ×200

Also, lung treated with DCMF, 300 mg/kg, revealed apparently normal tissue architecture, with thin-walled air-filled alveoli and normal alveolar sacs. The thin inter-alveolar septa had numerous capillaries and minimal connective tissue. The terminal bronchioles were lined by wavy ciliated simple cuboidal epithelium with underlying lamina propria, continuous musculosa layer and thin adventitia. Pulmonary blood vessels were mildly dilated (Fig. 11 and Table 8).

##### Immunohistochemical (TGF-B1) results

**Fig. 12:**
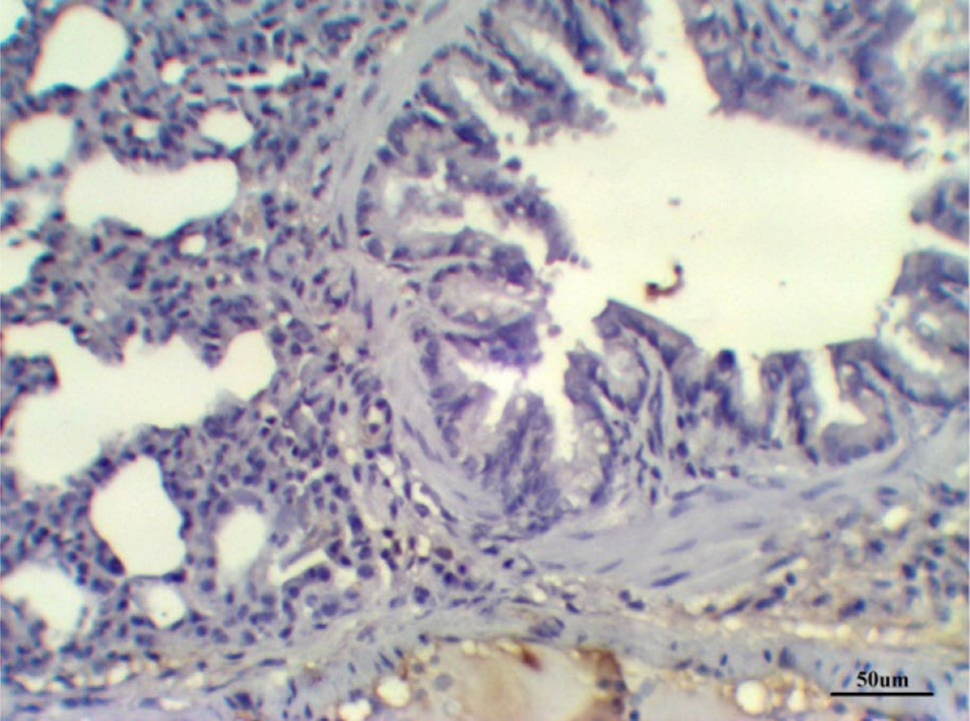
Lung control group stained with TGF-β1 immunohistochemical stain revealed −ve expression

**Fig. 13:**
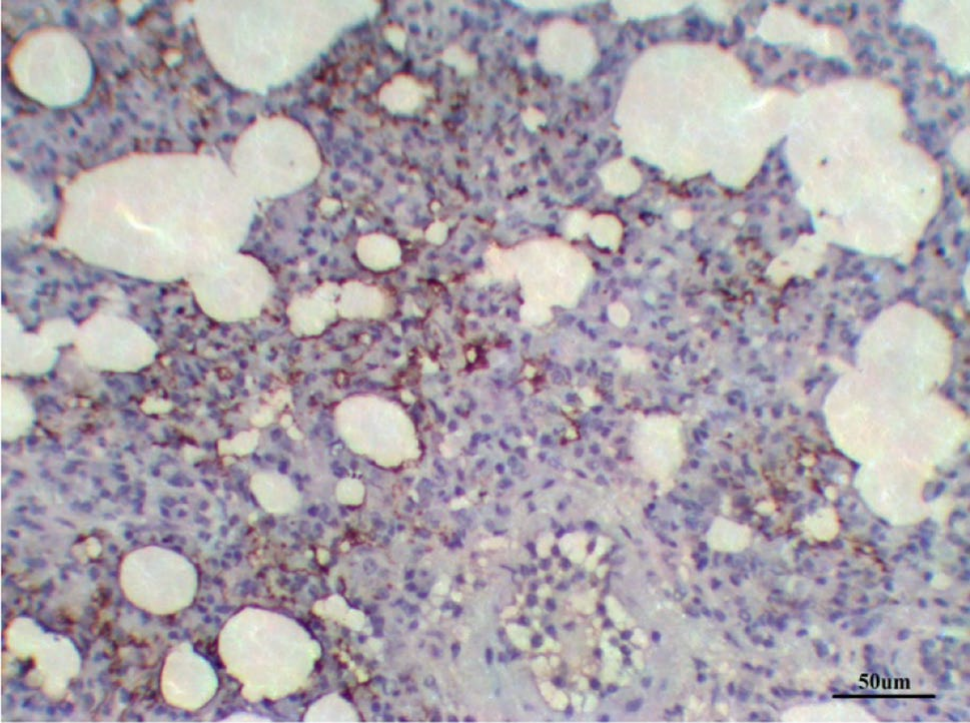
LPS group stained with TGF-β1 immunohistochemical stain revealed ++ve expression

**Fig. 14:**
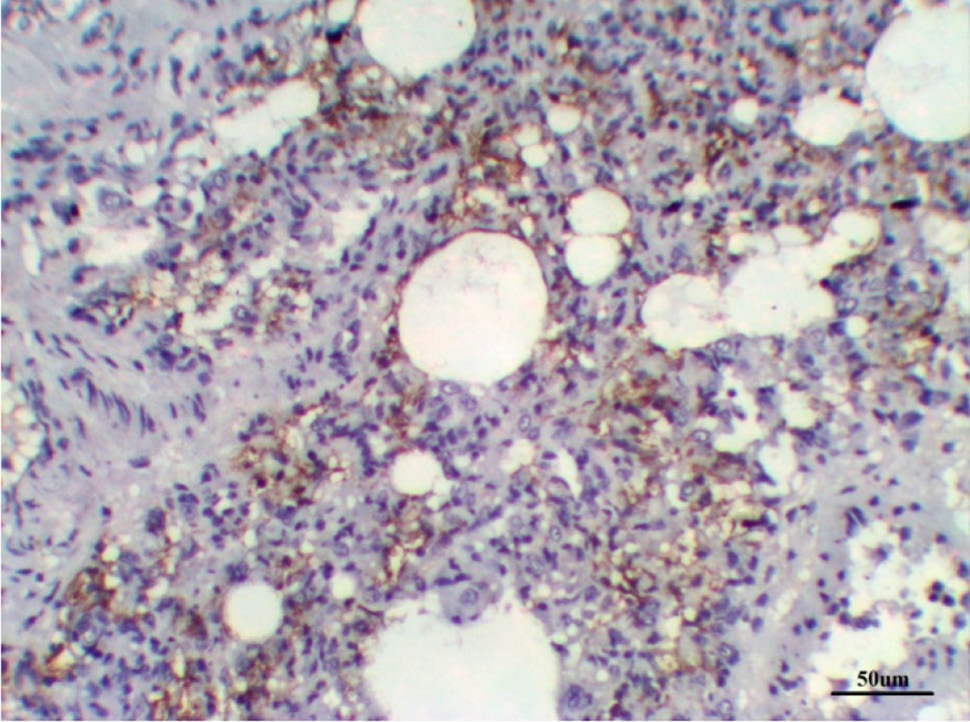
Lung LPS group treated with dexamethasone stained with TGF-β1 immunohistochemical stain revealed ++ve expression

**Fig. 15:**
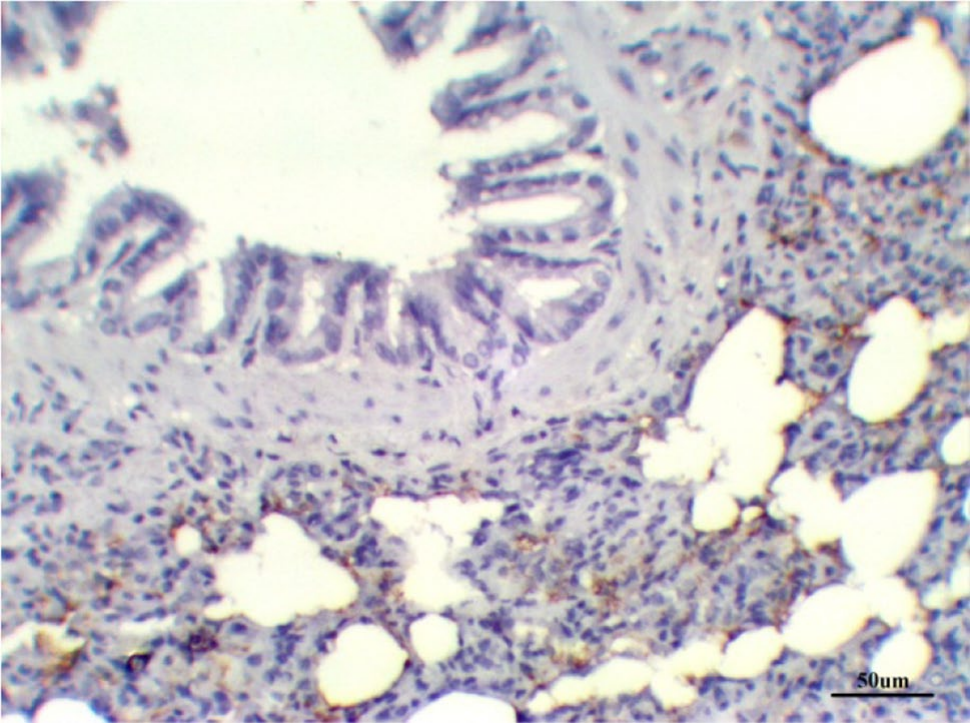
Lung LPS group treated with DCMF 200 mg/kg stained with TGF-β1 immunohistochemical stain revealed +ve expression

**Fig. 16:**
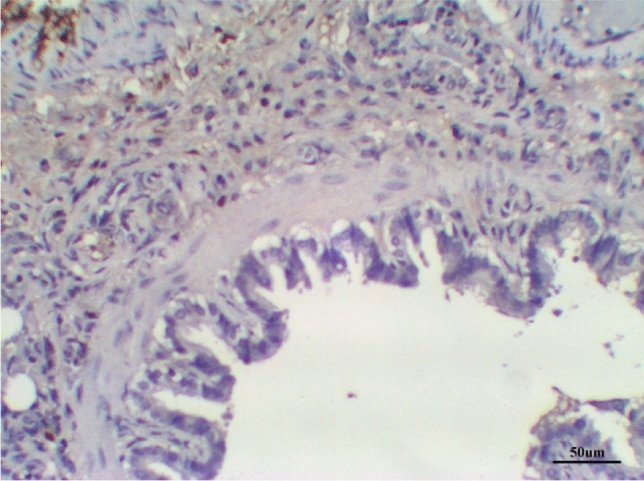
Lung LPS group treated with DCMF 300 mg/kg stained with TGF-β1 immunohistochemical stain revealed +ve expression

**Fig. 17:**
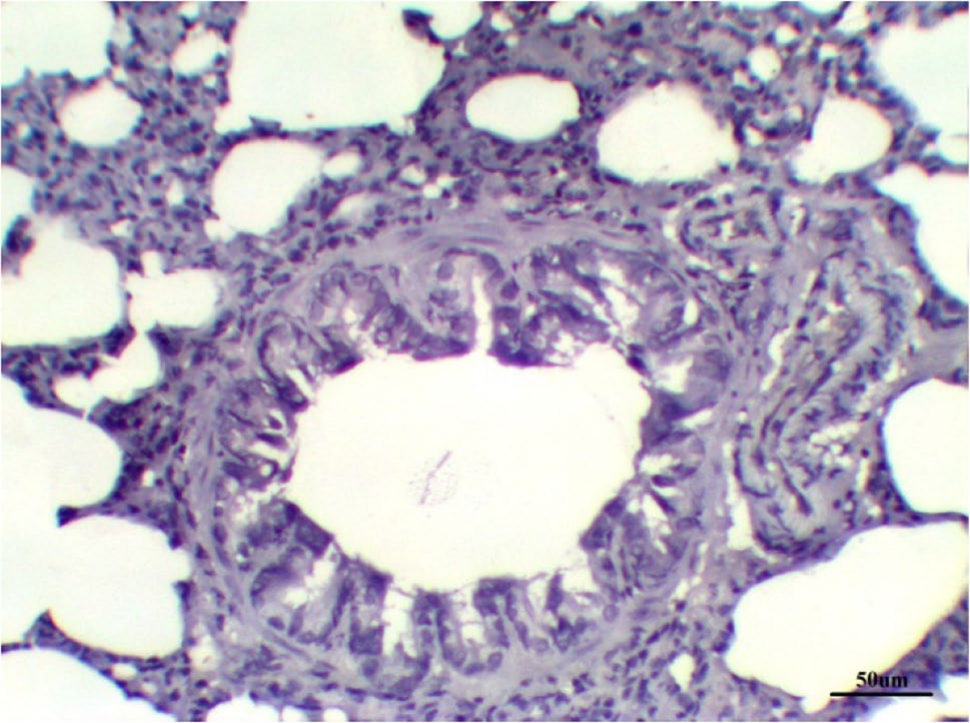
Lung treated with DCMF 200 mg/kg stained with TGF-β1 immunohistochemical stain revealed −ve expression

**Fig. 18:**
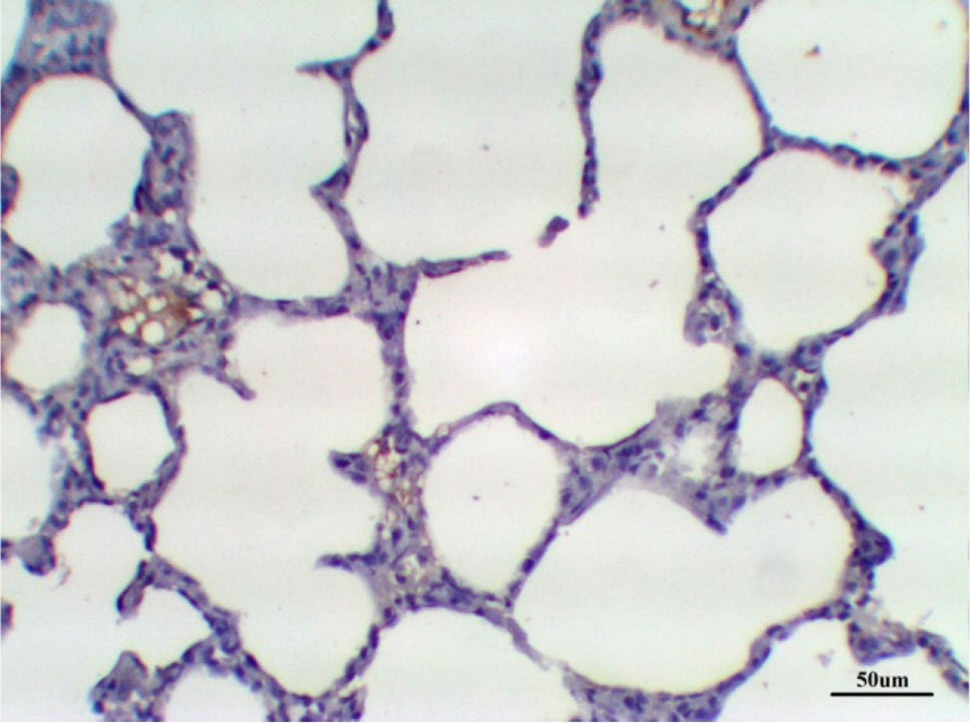
Lung treated with DCMF 300 mg/kg stained with TGF-β1 immunohistochemical stain revealed −ve expression

The lungs tissues of the control group, as well as the lungs of only treated groups with DCMF, 200 mg/kg treated and DCMF, 300 mg/kg, showed negative expression (−ve). The LPS (10 mg/kg) group and LPS group treated with dexamethasone (5 mg/kg) showed moderate expression (+ + ve). Meanwhile, the lung LPS group treated with DCMF (200 mg/kg bw) and the LPS (10 mg/kg bw ip) group treated with DCMF (300 mg/kg bw) treated group showed weak positive expressions (+ ve) (Figs. 12, 13, 14, 15, 16, 17, 18).

## Discussion

### *In-vitro* cyclooxygenases and lipoxygenase inhibitory activities

The arachidonic acid pathway plays a major role in inflammation and involves two metabolic pathways, the cyclooxygenase (COX) and the lipoxygenase (LOX) pathways (Chan et al. 2012). Non-steroidal anti-inflammatory drugs (NSAIDs) are the most used medication in these conditions with several side effects, including gastrointestinal irritation and renal toxicity (Bunimov and Laneuville 2008). Moreover, COX inhibitors may potentially increase the conversion of arachidonic acid to leukotrienes through the 5-LOX pathway due to substrate diversion, leading to the production of pro-inflammatory mediators and increasing the inflammatory response (Bunimov and Laneuville 2008; Bessone 2010). The resulting leukotrienes are pro-inflammatory mediators, and they could play an important role in increasing the inflammatory response associated with the usage of COX inhibitors. In the search for potential dual COX/LOX inhibitors, natural drugs serve as a useful source of potential compounds. Euphorbia species are used by traditional herbalists for the treatment of some inflammatory conditions such as dermatitis, conjunctivitis, and rheumatoid arthritis because of its unique chemical structure. *Euphorbia hirta* was established to inhibit the inflammatory effects of prostaglandin E2 on rabbit synovial fibroblasts (Chen et al. 2015). On the other hand, the diterpenoids isolated from the roots of *Euphorbia fischeriana* inhibited the production of inflammatory mediators such as prostaglandin E2, nitric oxide, IL-6 and TNF-α (Uto et al. 2012). *Euphorbia hirta* was proved to have substantially lower levels of pro-inflammatory cytokines (interferon-γ), IL-6, TNF-α and higher concentrations of anti-inflammatory cytokines (IL-4 and IL-5) (Ahmad et al. 2014). Diterpenes dominated metabolites in Euphorbia species were reported for their anti-inflammatory by inhibiting the generation of inflammatory cytokines (IL-1β, IL-6, and TNF-α) in addition to other inflammatory markers as iNOS, and COX-2 (Kemboi et al. 2021; Liu et al. 2014; Wang et al. 2021).

Bioactivity-guided fractionation revealed that DCMF showed higher activity amongst the tested samples, including the conventional standard drugs. Therefore, the identification of the chemical constituents responsible for the active fraction has become of great importance for the developing potential therapeutic agents. Based on previous literature surveys, terpenoids, coumarins, aromatic compounds, and steroids are the major secondary metabolites found in this genus. It has been reported that the terpenoid constituents in these plants may be responsible for their biological activities (Yener et al. 2019).

The detection of the chemical constituents present in herbal samples is an essential issue since the plant extracts have a variety of compounds with different chemical structures and complex matrices. Nowadays liquid chromatography–mass spectrometry (LC–MS) is currently the most widely used method for characterizing plant secondary metabolites (Selvi et al. 2018; Sun et al. 2018). In the last few decades, liquid chromatography–time-of-flight–mass spectrometry (LC-TOF–MS) has emerged as a sophisticated technique with fast scanning and high mass resolution properties (Huang et al. 2020). To the best of our knowledge, the present study may be the first report on the chemical profiles of TME and DCMF of *E. grantii* aerial parts. The results led to the tentative identification and characterization of 56 phytochemical compounds, with diterpenes being the predominant secondary metabolites in both samples. Diterpenoids identified genus *Euphorbia* can be classified into two types, higher and lower diterpenoids, based on their unique structure and taxonomic specificity. These types have different biosynthesis mechanisms that depend on the catalytic mechanism of the diterpenoid cyclase (classic type 1 and non-classic type 2) (Drummond et al. 2021; Zhao et al. 2022). In our samples, several classes of diterpenes were detected based on the fragmentation patterns, relevant literature, and MS data.

Overall, the findings of this study provide important insights into the potential biological activities of these plants and demonstrate the effectiveness of LC-TOF–MS in characterizing the complex chemical constituents of plant extracts. Further research can build upon these findings to explore the biological activities of the identified compounds and their potential use in medicine and other fields.

### Chemical profiling using LC-DAD-QToF-MS analysis

#### Tigliane type-diterpenes (phorbol and phorbol esters)

Five phorbol diterpenes have been identified from *E. grantii* (Fig. S1). The tentatively identified phorbol (**12**) is a tetracyclic diterpene derived from a hydride of tigliane (Wender and Rice 1997). Phorbol showed a molecular ion peak (MIP) in positive mode at *m/z* 387.1779 [M + Na]^+^ and MS^2^ fragment ions resulting from sequential loss of four –H_2_O molecules, indicating the presence of terminal hydroxy groups. Further loss of cyclohexanone moiety results *m/z* 195.0805 [M + H–4H_2_O–C_6_H_10_O]^+^ and followed by loss of –CH_2_O showed *m/*z 165.0694. In negative mode, phorbol showed a MIP at *m/z* 363.1810 [M − H]^−^ along with fragment ions at *m/z* 345.1708 [M − H–H_2_O]^−^, and 315.1598 [M − H–H_2_O–CH_2_O]^−^. The detection of the MIP and fragmentation pattern confirmed the tentative identification of phorbol (compound **12**) as shown in Table 3. The relative abundance of phorbol is less in methylene chloride extract compared to the methanolic extract (Buckingham 2020; Ghani and Badr 2020). The compound was isolated previously from *E. paralias* (Ghani and Badr 2020). Similarly, chromatographic peak at t_*R*_ 11.83 min shows MIP at *m/z* 413.1942 [M + Na]^+^ and corresponding fragments resulted from loss of water molecules and –CH_2_O (Table 3). This compound is tentatively identified as prostratin/12-deoxyphorbol-13-acetate (compound **19**) (Buckingham 2020; Tang et al. 2012) and has been previously isolated from *E. fischeriana* (Tang et al. 2012).

**Fig. 19:**
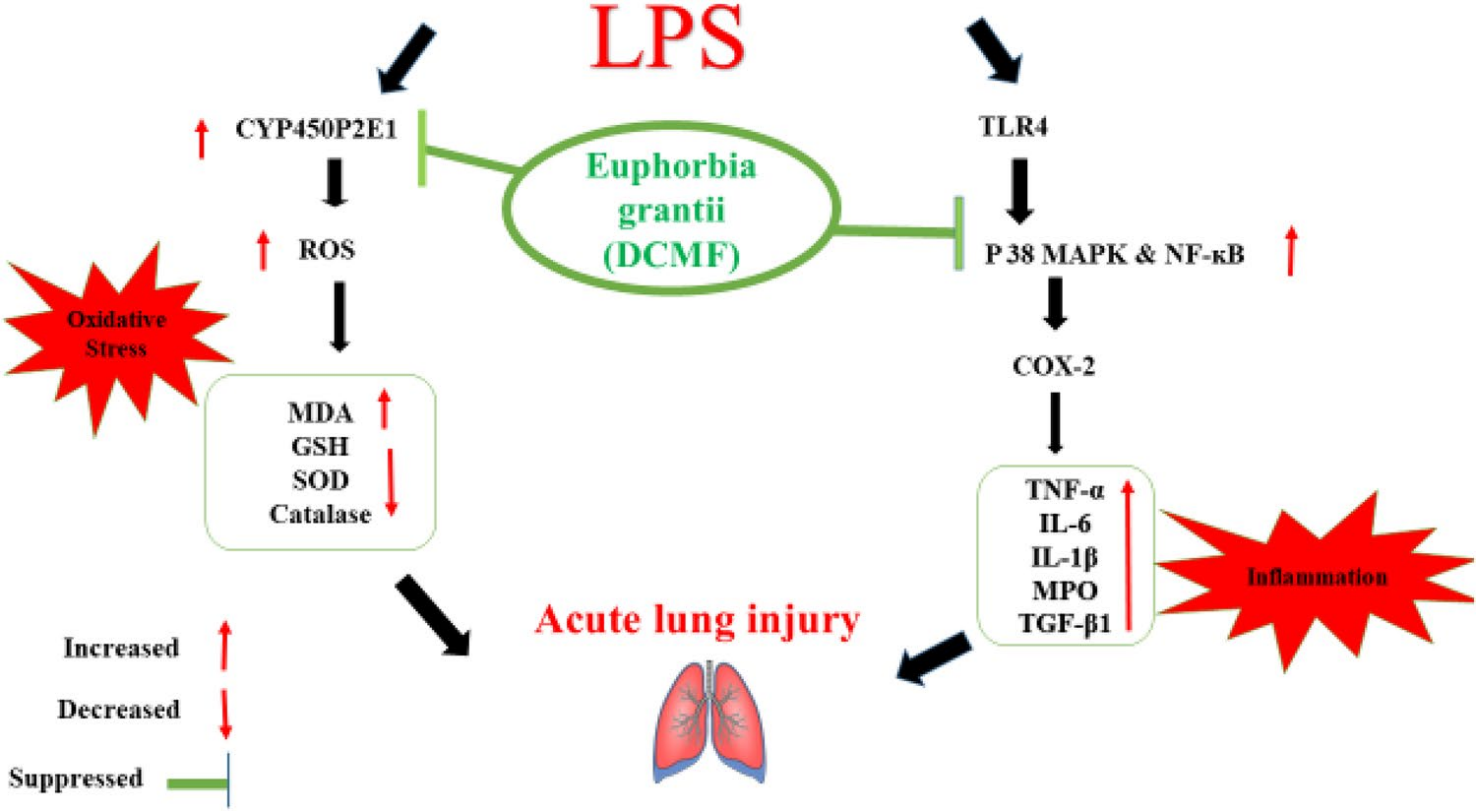
The potential effects of DCMF extract on LPS-induced acute lung injury in albino rats; the molecular mechanism of DCMF to defend the LPS-induced inflammation and oxidative stress in lung tissues by blocking the P38 MAPK, NF-ĸB pathway, and COX2; and inhibiting oxidative stress (ROS) by suppressing the cellular CYP450P2E1 gene expression

**Fig. 20:**
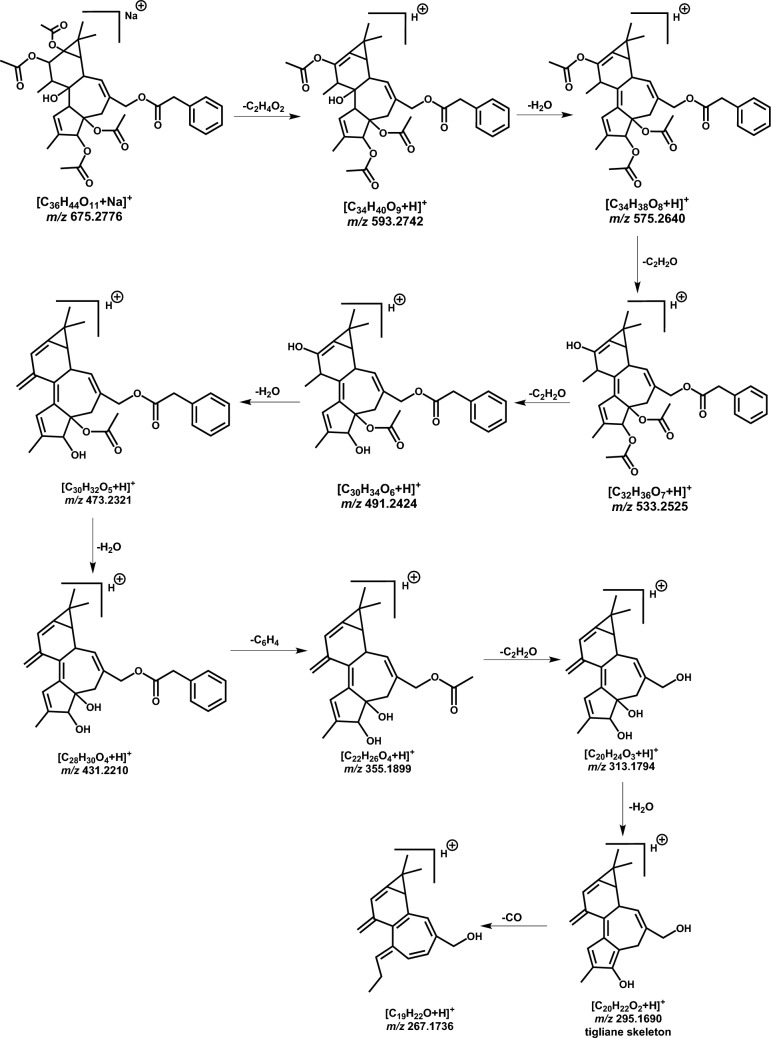
Tentative LC-ESI‐QToF fragmentation pattern of synagrantol A (tigliane skeleton) in positive ion mode

Further, deoxyphorbol ester compounds (isomers) observed at t_*R*_ 27.2 and 28.1 min (compounds **49** and **50**), respectively, had a MIP in positive mode at *m/z* 670.3224 [M + NH_4_]^+^. The MS^2^ fragment ions in positive mode showed sequential loss of acetate and water molecules respectively. A detailed fragmentation pathway was proposed in Figs. 19 and 20. Fragment ion peak of *m/z* 295.1690 formed based on the tigliane backbone skeleton produced due to the loss of terminal acetate, hydroxy groups, and loss of phenyl group along with –CO (electron withdrawing group). The proposed fragmentation correlates to synagrantol A (compounds **49** & **50**) (Fig. S1) (Buckingham 2020; Hassan et al. 2012). Chromatographic peak at t_*R*_ 28.0 min shows MIP at *m/z* 530.3117 [M + NH_4_]^+^ with molecular formula C_30_H_40_O_7_ which was tentatively identified as synagrantol B (**52**) (Table 3), based on its molecular features and previously published literature. Synagrantol A and B were isolated from *Synadenium grantii* (synonym *E. umbellata*) (Buckingham 2020; Hassan et al. 2012).

#### Lathyrane diterpenoids (ingol-type esters)

Ingol type diterpenes are derived from lathyrane diterpenes through oxygenated functional group at C-15 (15, 4-epoxide) (Fig. S2) (Vela et al. 2022; Livermore 2009). There are nine ingol-type diterpenes compounds tentatively identified in *E. grantii* (Fig. S3).

Chromatographic peaks at t_*R*_ 10 min and 12.8 min (compounds **15** & **16**) showed a similar MIP at *m/z* 367.2120 [M + H]^+^. The diagnostic fragment at *m/*z 349.2013, 331.1910, and 313.1804 resulted from sequential loss of water molecules followed by loss of carbonyl group resulting in *m/*z 285.1853. The fragment ion at *m/z* 197.1176 showed loss of two molecules of water and isopropenylphenol moiety [M + H–2H_2_O–C_9_H_10_O]^+^, which indicated a lathyrane type backbone skeleton. Compounds **15** and **16** were tentatively identified as ingol isomers (Buckingham 2020) (Fig. S3). Similarly, compounds **27, 32, 39, 42, 45, 47**, and **48** were tentatively identified under the class of lathyrane diterpenoids based on their MIP, molecular formula, corresponding fragment ions. Each compound’s fragment ions, along with putatively annotated loss of functional groups were detailed in Table 3 (Zhao et al. 2018).

#### Ingenane-type diterpenes (ingenol diterpene)

Only one ingenol diterpene was detected in *E. grantii* at t_*R*_ 13.3 min (**24**), and was identified as 17-hydroxyingenol (Buckingham 2020) (Fig. S4). This compound showed a MIP in negative mode at *m/z* 555.2447 [M + COOH]^−^. The MS^2^ fragment ion in the negative mode displayed a loss of C_6_H_6_O_4_ moiety, known as kojic acid, resulting in a fragment ion at *m/z* 367.2128 [M − H–C_6_H_6_O_4_]. Another fragment ion at *m/z* 359.1500 [M − H–C_6_H_14_O_4_]^−^ was produced due to loss of triethylene glycol moiety, followed by a fragment ion due to loss of carbonyl group at *m/z* 315.1593 [M − H–C_6_H_14_O_4_–CO_2_]^−^, succeeded by a fragment loss of an acetic acid moiety at *m/z* 255.1387 [M − H–C_6_H_14_O_4_–CO_2_–C_2_H_4_O_2_]^−^. 17-Hydroxyingenol (**24**) was previously isolated from *E. laurifolia* (Piaz et al. 2016).

#### Jatrophane diterpenes

Only one jatrophane diterpene was tentatively identified in *E. grantii* (compound **51**) at t_*R*_ 27.4 min, the compound was identified as serrulatin A (Fig. S5) (Buckingham 2020). The molecular base peak in positive mode was observed at *m/z* 719.3038 [M + Na]^+^. The ion fragment in positive mode was displayed by losing acetic acid moiety at *m/z* 637.3015 [M + H–C_2_H_4_O_2_]^+^, followed by a loss of ion fragment of hydroxy benzoyl moiety at *m/z* 559.2908 [M + H-C_7_H_6_O_3_]^+^, then it was followed by fragment ions with a loss of acetic acid moieties, propene moiety, and water molecule. The deprotonated fragment ion found at *m/z* 181.1215 was assigned for [C_11_H_16_O_2_ + H]^+^. Details about the observed fragments and their corresponding fragment ion losses were mentioned in Table 3. Serrulatin A was previously isolated from *E. serrulata* (Hohmann et al. 2000).

#### Atisane diterpenes

Compounds **20** and **21** observed at t_*R*_ 12.2 min and 12.8 min, respectively, were identified as atisane-type diterpene. These compounds were tentatively identified as trihydroxy-3-atisanone isomers (Fig. S6) (Buckingham 2020). A molecular base peak in positive ion mode was detected at *m/z* 337.2366 [M + H]^+^ and a MIP in negative mode was observed at *m/z* 381.2283 [M + COOH]^−^. MS^2^ fragment ions (positive mode) showed a loss of water molecules resulted in fragment ions at *m/z* 319.2265, 301.2159, and 283.2052. In addition to fragment ions showed a loss of pentanol moiety, acetyl moiety, and methylene moieties. The least fragment ions were observed at *m/*z 175.1113 [M + H–C_5_H_12_O–C_2_H_2_O–CH_2_–H_2_O]^+^ and *m/z* 133.1004 [M + H–C_5_H_12_O–2C_2_H_2_O–CH_2_–H_2_O]^+^, which confirmed the presence of atisane type backbone skeleton. Compounds with similar skeleton (atisan-3-one) were previously isolated from *Sapium insigne* (Yan et al. 2018).

#### Daphnane-type diterpenes (resiniferonoids)

Resiniferonoid (daphnane-type diterpenoids) (Jin et al. 2019) was observed at chromatographic peak at t_*R*_ 11.4 min (compound **18**) and was tentatively identified as euphopiloside A (Fig. S7) (Buckingham 2020). This compound showed a MIP in positive mode at *m/z* 533.2362 [M + Na]^+^, and the fragment ion observed were due to sequential loss of water molecules, CH_2_ moiety, and C_2_H_2_O_2_ moiety resulted in *m/z* 493.2430 [M + H-H_2_O]^+^, 475.2334 [M + H-2H_2_O]^+^, 449.2173 [M + H–2H_2_O–CH_2_]^+^, 391.2114 [M + H–2H_2_O–CH_2_–C_2_H_2_O_2_]^+^. An independent ion fragment found at *m/z* 209.1528 [C_13_H_20_O_2_ + H]^+^. In addition, a MIP in negative mode was shown at *m/z* 509.2392 [M − H]^−^ and the fragment ion in the negative mode was observed at *m/z* 343.2124 [M − H–C_8_H_6_O_4_]^−^. Euphopiloside A was previously isolated from *E. pilosa* (Kemboi et al. 2021; Zhang et al. 2014).

#### Myrsinol-type and premyrisane type diterpenes

Dehydroeuphoreppinol (t_*R*_ 8.8 min, compound **14**) was the first identified diterpene belonging to premyrsinane diterpene isolated from *E. grantii* is (Fig. S8) (Buckingham 2020). This compound showed a MIP in negative mode at *m/z* 427.1974 [M + COOH]^−^. The hydrogenated structure of euphoreppinol, which has been isolated from *E. aleppica* (Shit et al. 1998), is similar to dehydroeuphoreppinol. Further, chromatographic peak at t_*R*_ 22.1 min (compound **33**) was tentatively identified as euphoppin A (Buckingham 2020). The corresponding MIPs at *m/z* 610.3220 [M + NH_4_]^+^ and 615.2771 [M + Na]^+^ were observed in the positive ion mode. While in the negative ion mode a MIP was observed at *m/z* 637.2866 [M + COOH]^−^. The fragment ion at *m/z* 577.2682 [M − H–CH_2_]^−^ in the negative mode was observed as a result of CH_2_ moiety loss, followed by a loss of methyl alcohol at *m/z* 545.2374, proceeded by a loss of C_7_H_8,_ denoting the seven membered carbon ring of the premyrsinane system at *m/z* 381.1741, along with a loss of formaldehyde moiety at *m/z* 351.1636. This compound was identified as euphoppin A, previously isolated from *E. aleppica.* (Yang et al. 1995). Similarly, compounds **38** and **46** were tentatively identified under the class of premyrisane type diterpenes. The fragment ions and corresponding loss of fragments were shown in Table 3 (Wang et al. 2015).

#### Myrsinane-type diterpenes

Myrsinane-type is a tricyclic diterpenes, the first compound has myrsinane skeleton was tentatively identified as decipinone C (Fig. S9) (Buckingham 2020). Chromatographic peaks at t_*R*_ 19.5 min, 20.2 min, 21.1 min, and 21.9 min correspond to compounds **28–31**. Similar MIP in positive mode at *m/z* 601.2619 [M + Na]^+^, the fragmentation pattern in the positive ion mode displayed fragment ions as a result of water loss at *m/z* 561.2696 and 543.2602. Further loss of acetic acid moiety, water molecule, acetyl, and ethylene moieties, resulted in *m/z* 519.2590, 501.2481, 459.2377, 431.2088, 413.1960, 371.1854, 353.1750, 311.1638, 293.1535, 265.1583, and 223.1111 (Table 3). The least fragment ions were observed at *m/*z 195.1161 and 181.1007, which explain the backbone skeleton of myrsinane diterpenes. The pattern showed loss of three acetyl groups and a butanoyl group fragmented into acetyl, ethylene moiety, and CH_2_ moiety, which matched with the decipinone C substituent (Fig. S9). This compound was isolated from *E. decipiens* (Zahid et al. 2001). Similarly, compounds **40–41** and **43–44** were tentatively identified under myrsinane type diterpenes based on their extract mass, corresponding fragment ions, and molecular formulas (Vasas and Hohmann 2014).

#### Ent-abietane-type diterpenes

Chromatographic peak at t_*R*_ 8.7 min (compound **13**) was tentatively identified as euphelionolide A or isomers euphelionolide B/C/D/E/F/G/L, a tricyclic diterpenes of ent-abietanes containing lactone ring (Buckingham 2020; Bicchi et al. 2001; Su et al. 2003) (Fig. S10). The peak has shown only a molecular base peak in the negative mode at *m/z* 393.1919 [M + COOH]^−^. The compound and it’s isomers were isolated before from *E. helioscopia* (Wang et al. 2018). Further, chromatographic peak at t_*R*_ 12.5 min and 13.9 min (compounds **22** and **23**) were tentatively described as euphelionolide N or isomers euphelionolide I/H (Buckingham 2020) (Fig. S10). It has a molecular base peak in positive mode at *m/z* 347.1853 [M + H]^+^ and MS^2^ fragmentation ions in the positive mode at *m/z* 329.1743, 315.1950 resulted from the loss of water molecule and -CH_2_. Further fragmentation showed the loss of iso-methyl lactone moiety, oxygen atom, and ethenol (vinyl alcohol) moieties at *m/z* 267.1587 [M + H–C_5_H_4_O]^+^, 251.1629 [M + H–C_5_H_4_O–O]^+^, and 207.1368 [M + H–C_5_H_4_O–O–C_2_H_4_O]^+^, respectively. The MIP in negative mode adduct was shown at *m/z* 391.1764 [M + COOH]^−^ and fragmentation ion are displayed at *m/z* 289.1805 [M − H–H_2_O–C_2_O_2_]^−^. Similarly, compound **26** was identified as euphelionolide J or its isomer euphelionolide K. In addition, jolkinolide B (compound **25**) was identified from *E. grantii* and has ent-abietane skeleton (Buckingham 2020; Su et al. 2003) (Fig. S11).

#### Miscellaneous compounds

##### Monoterpenoids

Chromatographic peak at t_*R*_ 5.6 min (compound **7**) showed MIP in negative mode at *m/z* 227.0925 [M − H]^−^ and a fragment ion at *m/z* 183.1024 [M − H–CO_2_]^−^. The compound was tentatively identified as loganetin (Buckingham 2020), a known irridoid monoterpene previously isolated from *Scaevola taccada (Euphorbiaceae)* (Yeshi et al. 2022; Suthiwong et al. 2017). Another monoterpene identified at t_*R*_ 10.26 min (compound **17**). A MIP in positive mode was observed at *m/z* 197.1172 [M + H]^+^ and a fragment ion in the positive mode was displayed at *m/z* 179.1029 [M + H–H_2_O]^+^. The compound was identified as loliolide (**47**) and previously isolated from *E. supina* (Tanaka and Matsunaga 1989) (Fig. S12).

##### Sesquiterpenoids

Compound **8** (t_*R*_ 5.7 min) displayed a MIP adduct in the positive mode at *m/z* 429.2095 [M + Na]^+^ and a MIP in the negative mode adduct at *m/z* 209.1535 due to loss of glucose moiety [M + H–C_6_H_12_O_6_–H_2_O]^+^, followed by a further fragmented ion at *m/z* 167.1066 [M + H–C_6_H_12_O_6_–H_2_O–C_3_H_6_]^+^ due to loss of a water molecule and propene moiety. The compound was identified as euphorbioside B (Buckingham 2020) (Fig. S13). Compound **11** (t_*R*_ 7.6 min) showed a MIP in positive mode at *m/z* 422.2385 [M + NH4]^+^. Fragment ions in the positive were displayed at *m/z* 227.1638 [M + H–C_6_H_10_O_6_]^+^, 209.1528 [M + H–C_6_H_10_O_6_–H_2_O]^+^, and 183.1011 [M + H–C_6_H_10_O_6_–C_3_H_8_]^+^, respectively. In negative mode, MIP found at *m/z* 449.2028 [M + COOH]^−^ and fragment ions at *m/z* 215.1288 [M − H–C_8_H_12_O_5_]^−^, 203.0927 [M − H–C_10_H_16_O_4_]^−^, 157.0506 [C_7_H_8_O_4_–H]^−^, and 113.0608 [C_7_H_8_O_4_–H–CO_2_]^−^ were also observed. Compound **11** was tentatively described as euphorbioside A (Buckingham 2020) (Fig. S13). Euphorbioside A and B were previously isolated from *E. resinifera* (Fattorusso et al. 2002).

##### Triterpenoids

Three triterpenoids were identified in *E. grantii*, compound **54** (t_*R*_ 31.1 min) having MIP in positive mode at *m/z* 429.4091[M + H]^+^ and identified as friedelinol. Compounds **55** and **56** (t_*R*_ 34.7 and 35.1 min), both displayed a similar MIP in the negative mode at *m/z* 425.3755 [M − H]^−^, which were tentatively identified as friedelin or euphol (Fig. S14). These compounds were previously isolated from *S. grantii* (*E. umbellate*) (Munhoz et al. 2014).

### Biological assessment

Despite extensive studies on the pathogenesis of ALI, no effective treatment has been developed to date (Chen et al. 2015). Therefore, it is important to explore effective medications and innovative treatments. Lipopolysaccharide (LPS), a component of the Gram-negative bacterial cell membrane, is widely used to study the pathogenesis and prevention of ALI in mice due to its ability to induce the inflammatory response and immune dysfunction (Liu et al. 2014). LPS-induced ALI is characterized by a neutrophilic inflammatory response, inflammatory mediator release, and pulmonary edema. Pro-inflammatory cytokines released in the early phase of an inflammatory response, play a critical role in ALI, and impact to the severity of lung injury (Meduri et al. 1995). TNF-α, a crucial cytokine in ALI, has been found to be elevated in patients with ALI or ARDS (Cornélio Favarin et al. 2013). IL-1β plays an important role in the progression of ALI as it can inhibit fluid transport through the distal lung epithelium, trigger surfactant abnormalities, and boost protein permeability through the alveolar–capillary barrier (Wei and Huang 2014). Additionally, IL-6 is a marker of endotoxin-induced ALI (Wei and Huang 2014). These cytokines initiate, amplify, and perpetuate the inflammatory cascade in ALI/ARDS (Meduri et al. 1995a, 1995b). Moreover, these cytokines not only increase the inflammatory response and cause inflammatory injury but they also recruit neutrophils into the lung and increase the MPO activity in the lung tissues (Wei and Huang 2014). MPO, a major constituent of neutrophil cytoplasmic granules, is a key characteristic of the neutrophil infiltration (Qiu et al. 2011). In this study, we found that DCMF significantly attenuated the production of pro-inflammatory cytokines, such as TNF-α, IL-6, IL-β1, and MPO, which significantly increased in the LPS-treated group after 4 h of intraperitoneal injection on the 7th day. Therefore, the results indicated that the protective effects of *E. grantii* on LPS-induced ALI may be attributed to the inhibition of inflammatory cytokines. Several Euphorbia plant species have been used as therapeutics in various traditional medicine systems, particularly the Euphorbia diterpenoids, which have pharmacological roles in suppressing pro-inflammatory cytokines such as TNF-α and IL-6 (Kim et al. 2018; Su et al. 2019). Besides that, Zhang et al. report that diterpenoid has a potent mechanism for reducing the cytokines IL-6 and IL-1β in vivo as well as regulating the protein concentrations of iNOS, NF-κβ, and phosphorylated IB, which is consistent with our findings (Zhang et al. 2019).

Oxidative stress is defined as a status of an imbalance between cellular anti-oxidative capacity and reactive oxygen species formation, resulting from the dysregulation of antioxidant system. Therefore, enhancing cellular antioxidant capacity or scavenging reactive oxygen species may ameliorate this imbalance and have a positive impact on a variety of pathological and disease disorders (Matsebatlela et al. 2015). Antioxidant enzymes and non-oxidants play a crucial role in the defense against free radicals such as O_2_ and HO, as any accumulation of these free radicals caused by LPS-induced oxidative stress can stimulate inflammatory cytokines (Shokry et al. 2022; El-Shiekh et al. 2023; Eloutify et al. 2023; Salem et al. 2023; El-Shiekh et al. 2021). MDA is a reliable indicator of oxidative stress and is used to reflect the level of cell damage caused by reactive oxygen metabolites (Ahmed 2015). Endogenous anti-oxidative molecules, including SOD, GSH, and CAT had critical roles in the defense against reactive oxygen species activated by oxidative stress injury (Jing et al. 2015; Kim et al. 2014). Our treatments had the pharmacological ability to regulate the oxidant and antioxidant enzymes (GSH, MDA, SOD, and CAT), which also revealed the ability of *E. grantii* to mitigate the side effects of LPS on lung cells.

LPS stimulates its inflammatory reaction through the activation of TLR4 signaling pathways to control the release of pro-inflammatory cytokines (Kagan and Medzhitov 2006). Activation of TLR4 by LPS stimulates the activation of NF-κB signaling pathways. NF-κB is an important regulators of pro-inflammatory gene expression, regulating the expression of inflammatory-related cytokines (Yadav et al. 2003; Chun et al. 2007). Under normal situations, NF-κB is present in its inactive cytoplasmic form bound to the repressor of NF-κB (IκBs). However, once stimulated by LPS, the degradation and phosphorylation of IκB-α will amplify, resulting in the release of free NF-κB p65 and translocation from the cytoplasm to the nucleus, followed by the transcription of specific target genes, such as TNF-α, IL-1β, and IL-6 (Bouwmeester et al. 2004; Wilson et al. 1999). The signaling pathway of MAPKs plays an important roles in regulating cytokines production (Zou and Shankar 2015; Nick et al. 1996). To illustrate the inhibitory effect of *E. grantii* on cytokines production, we examined the effects of DCMF on the expression of NF-κB and MAPK activation. The results showed that the DCMF inhibited the expression of NF-κB and MAPK activation induced by LPS. Previously, diterpenes of Euphorbia were found to reduce the formation of inflammatory factors and decreasing the expression of NF-κB (Wang et al. 2020).

Furthermore, changes in pro-inflammatory cytokines such as TNF-α and IL-6 have been shown to correlate with changes in Cytochromes P450 (CYP) expression and enzymatic activity during infection and inflammation (Vizzini et al. 2021). In this animal model, the results were consistent with LPS treatment of rats inducing *CYP2E1* expression, whears rats administered orally DCMF of *E. grantii* inhibited the inducible *CYP* expression levels during the inflammation.

TGF-β1 is an immunomodulatory cytokine that regulates the proliferation and differentiation of various cell types. It also contributes to the maintenance of tissue architecture by influencing the production of extracellular matrix components (Magnan et al. 1994). Additionally, it has been shown to play a role in controlling inflammation, mucus hypersecretion, and excessive fibrosis of airway tissues in chronic airway diseases, such as chronic rhinosinusitis, asthma, and chronic obstructive pulmonary disease (André et al. 2015; Sejima et al. 2011). TGF-β1 is implicated in most of the cellular processes toward airway remodelling, subepithelial fibrosis, airway smooth muscle remodelling, epithelial changes, and microvascular changes in asthmatic patients (Sejima et al. 2011; Kim et al. 2007; Halwani et al. 2011). According to Kang et al. (2007), TGF-β1-induced fibrosis and apoptosis contribute to the pathogenesis of a wide variety of pulmonary and extrapulmonary diseases and disorders. Our results revealed that the rats administered orally DCMF of *E. grantii* downregulated the levels of TGF-β1 during the inflammation.

Several Euphorbia species, particularly those containing Euphorbia diterpenoids, have been used in various traditional medicine systems as therapeutics with pharmacological roles in suppressing pro-inflammatory cytokines such as TNF-α, IL-6, IL-1β, iNOS, and NF-κβ (Zhang et al. 2019; Choodej et al. 2020). Evaluation of the water fraction of *E. royleana* latex showed dose-dependent anti-arthritic and anti-inflammatory activities in acute and chronic test models in mice and rats by reducing the migration of leukocytes in addition to dose-related peripheral analgesic effects (Bani et al. 2000). Phorbol esters, members of the tigliane family of diterpenes and isolated from plants belonging to the family Euphorbiaceae, are reported to have anti-inflammatory effects role by inhibiting proinflammatory cytokines (IL-8) (Vazquez et al. 1999). Lathyrane diterpenoids from *E. lathyris* were investigated for the inhibition activities against induction of NO generation by LPS in murine macrophage cells (RAW264.7). It was found that they exerted anti-inflammatory activity via reducing the production of cytokines and the expression of proteins phosphorylated nuclear factor kappa light polypeptide gene enhancer in B-cells inhibitor, alpha (IκBα), nitric oxide (NO) production, inducible nitric oxide synthase (iNOS), and NF-κB (Zhang et al. 2019). Jatrophane polyesters isolated from the leaves of *Euphorbia peplus* Linn. were investigated for their anti-inflammatory effects induced by LPS in RAW264.7 macrophage cells and they markedly inhibited NO production in LPS-induced RAW264.7 cells (Li et al. 2022). NO inhibitory diterpenoids from euphorbia as potential anti-inflammatory agents was highly documented (Wei et al. 2019; Su et al. 2019; An et al. 2019; Wan et al. 2016), also for their NF-κB signaling inhibition (Tran et al. 2017). Based on these results, it was determined that this diterpenoid could be a potential anti-inflammatory agent for future studies.

In summary, our results provide evidence that *E. grantii* is an effective anti-inflammatory plant and its respective DCMF was active both in vitro and in vivo. In the rat model of lung inflammation, DCMF activity was as the steroid drug dexamethasone having the ability to reduce the inflammatory cytokines after LPS-induction of ALI. Also, it abolished the accumulation of oxidative stress biomarkers.

## Conclusions

Our results reflected the pharmacological activity of the DCMF (200 mg/kg and 300 mg/kg) that exhibited the remarkable protective effects on ALI via the attenuations of inflammatory cytokines (TNF-α, IL-6, IL-β1 and MPO) and oxidative stress biomarkers (MDA), which are the side effects of LPS. Administration of DCMF (300 mg/kg b. wt.) elevated the antioxidant enzymes (SOD, catalase, and GSH). Furthermore, the underlying mechanism was possibly associated with MAPK, CY450P2E1, and NF-κB pathways, as indicated by the results of gene expression, and western blots. According to the histopathological results, the oral administration of DCMF (300 mg/kg b. wt.) showed a significant amelioration of the side effects of LPS in lung tissue compared to other groups. In addition, the fraction showed promising activity by inhibition of COX-1, COX-2, and LOX using in vitro kits. Using LC/MS, 59 metabolites were identified, where the diterpenes were the dominant metabolites (34 compounds) in the active fraction. Finally, this study reported for the first time that *E. grantii* diterpenes provide interesting agent to develop novel anti-inflammatory drug.

## Future research directions

The use of glucocorticoids is limited by their side effects, and there is a great need to develop new agents with the anti-inflammatory potency of standard glucocorticoids but with fewer side effects. Our study provided evidence for the potential development of *E. grantii* as an efficient and therapeutic drug against ALI in the future. We can test its effects spanning different mechanisms such as interfering with most inflammatory pathways and suppressing inflammation in a variety of diseases including inflammatory response associated with many acute and chronic inflammatory diseases as rhinitis, asthma, multiple sclerosis, rheumatoid arthritis, and conjunctivitis. Further comprehensive studies exploring the clinical applications, formulations, and interactions of *E. grantii*. In addition, future directions could be to track the potential of *E. grantii* as widely and successfully agent used in the treatment of inflammatory diseases with minimal systemic side effects as reflected from acute and chronic toxicity studies. Also, the identification and isolation of bioactive compounds and the description of the mechanism of action of these compounds in living systems should be investigated in the future. Consequently, biologically active compounds obtained could be exploited on an industrial scale.

### Supplementary Information

Below is the link to the electronic supplementary material.

Supplementary file1 (DOCX 286 KB)

## Data Availability

The authors declare that the data supporting the findings of this study are available within the paper and its Supplementary Information files. Should any raw data files be needed in another format they are available from the corresponding author upon reasonable request. Source data are provided with this paper.

## Abbreviations

ALI: Acute lung injury
APCS: Antigen-presenting cells
ARDS: Acute respiratory distress syndrome
CAT: Catalase
COX-1: Cyclooxygenase 1
COX-2: Cyclooxygenase 2
CY450P2E1: Cytochrome P450 family 2 subfamily E member 1
ELISA: Enzyme linked immunosorbent assay
GSH: Reduce glutathione
IL-1: Interleukin-1
IL-6: Interleukin-6
LC-DAD-QToF: Liquid chromatography diode array detector-quadrupole time-of-flight mass spectrometry
LOX: Lipoxygenase
LPS: Lipopolysaccharide
MDA: Malondialdehyde
MPO: Myeloperoxidase
NF-κB p65: Nuclear factor Kappa B
p38.MAPK14: Mitogen-activated protein kinase 14
SOD: Superoxide dismutase
TGF-β1: Transforming growth factor-beta 1
TLR4: Toll-like receptor 4
TNF-α: Tumor necrosis factor-α
