# Supplementary material for: LC-QToF chemical profiling of Euphorbia grantii Oliv. and its potential to inhibit LPS-induced lung inflammation in rats via the NF-κB, CY450P2E1, and P38 MAPK14 pathways
Source: Inflammopharmacology. 2023 Aug 12;32(1):461–94. doi: 10.1007/s10787-023-01298-7 (PMC10907465; doi:10.1007/s10787-023-01298-7)
Supplement: Supplementary file 1 — Supplementary file1 (DOCX 286 KB) [file 10787_2023_1298_MOESM1_ESM.docx]

|  | | | | | |
| --- | --- | --- | --- | --- | --- |
| **A: Phorbol esters** | | | | | |
| **#** | **R_1_** | **R_2_** | **R_3_** | **R_4_** | **Compound name** |
| **12** | OH | OH | OH | OH | Phorbol |
| **19** | H | *O*-acetyl | OH | OH | Prostratin |
| **52** | *O*-tiglyl | *O*-tiglyl | H | OH | Synagrantol B |
| **B: Phorbol acetyl ester** | | | | | |
| **49-50** | *O*-acetyl | *O*-acetyl | *O*-phenyl acetyl | *O*-acetyl | Synagrantol A |

**Fig. S1.** The chemical structures of phorbol and deoxyphorbol esters identified in dichloromethane fraction and total methanolic extract of *E. grantii.*

**Fig. S2.** Ingol-lathyrane type diterpenes skeleton

|  | | | | | |
| --- | --- | --- | --- | --- | --- |
| **Ingol diterpenes** | | | | | |
| **#** | **R_1_** | **R_2_** | **R_3_** | **R_4_** | **Compound name** |
| **15** | OH | OH | OH | OH | Ingol |
| **24** | OH | OH | *O*-tiglyl | OH | 4,15-Epoxy-3,7,8,12-tetrahydroxy-5-lathyren-14-one; (2β,3β,4β,5E,7α,8α,12α,13α,15β)-form, 8-Tigloyl |
| **26** | *O*-acetyl | *O*-acetyl | *O*-acetyl | *O*-acetyl | 4,15-Epoxy-3,7,8,12-tetrahydroxy-5-lathyren-14-one; (2β,3β,4β,5E,7α,8α,12α,13α,15β)-form, Tetra-Ac isomers |
| **28** | *O*-acetyl | *O*-phenyl acetyl | *O*-acetyl | *O*-acetyl | 4,15-Epoxy-3,7,8,12-tetrahydroxy-5-lathyren-14-one; (2β,3β,4β,5E,7α,8α,12α,13α,15β)-form, 7-O-(Phenylacetyl), 3,8,12-tri-Ac |
| **30** | OH | *O*-phenyl acetyl | *O*-acetyl | *O*-acetyl | Euphorblin G |
| **32** | *O*-acetyl | OH | *O*-tiglyl | *O*-acetyl | 4,15-Epoxy-3,7,8,12-tetrahydroxy-5-lathyren-14-one; (2β,3β,4β,5E,7α,8α,12α,13α,15β)-form,8-Tigloyl, 3,12-di-Ac |
| **34** | *O*-acetyl | *O*-(4-hydroxyphenyl acetyl) | *O*-acetyl | *O*-acetyl | Euphorblin H |
| **36** | *O*-acetyl | *O*-acetyl | -*O*-(2-methylbutanoyl) | *O*-acetyl | 4,15-Epoxy-3,7,8,12-tetrahydroxy-5-lathyren-14-one; (2β,3β,4β,5E,7α,8α,12α,13α,15β)-form, 8-O-(2-Methylbutanoyl), 3,7,12-tri-Ac isomers |
| **40** | *O*-benzoyl | OH | *O*-tiglyl | *O*-acetyl | 4,15-Epoxy-3,7,8,12-tetrahydroxy-5-lathyren-14-one; (2β,3β,4β,5E,7α,8α,12α,13α,15β)-form, 3-Benzoyl, 8-tigloyl, 12-Ac isomers |

**Fig. S3.** The chemical structures of ingol and ingol esters determined in the dichloromethane fraction and total methanol extract of *E. grantii*.

**Fig. S4.** Chemical structure of 17-hydroxyingenol; 20-deoxy, 3-*O*-β-D-glucopyranoside.

**Fig. S5.** Chemical structure of serrulatin A (8,14,15-triacetoxy-3-benzoyloxy-6,9-epoxy-9-hydroxy-7-tigloyloxyjatropha-4*Z*,11*E*-diene).

**Fig. S6.** Chemical structure of trihydroxy-3-atisanone.

**Fig. S7.** Chemical structure of euphopiloside A.

|  | | | | | | | |
| --- | --- | --- | --- | --- | --- | --- | --- |
| **Type A** | | | | | | | |
| # | **R_1_** | **R_2_** | **R_3_** | **R_4_** | **R_5_** | **R_6_** | **Compound name** |
| **14** | OH | OH | OH | OH | C=O | OH | Dehydroeuphoreppinol |
| **33** | *O*-acetyl | *O*-acetyl | *O*-acetyl | *O*-tiglyl | OH | OH | Euphoppin A |
| **46** | *O*-acetyl | *O*-acetyl | *O*-tiglyl | *O*-acetyl | *O*-acetyl | *O*-acetyl | Euphoreppine A |
| **Type B** | | | | | | | |
| **38** | *O*-propionyl | *O*-benzoyl | *O*-acetyl | *O*-acetyl | OH | OH | Euphordraculoin K |

**Fig. S8.** Chemical structures of premyrsinane type diterpenes present in *E. grantii*.

|  | | | | |
| --- | --- | --- | --- | --- |
| **Type A** | | | | |
| # | **R_1_** | **R_2_** | **R_3_** | **Compound name** |
| **28-31** | *O*-acetyl | *O*-butanoyl | OH | Decipinone C |
| **Type B** | | | | |
| **40-41** | *O*-iso butanoyl | *O*-acetyl | *O*-acetyl | Euphorbiaproliferin C |
| **43-44** | *O*-propanoyl | *O*-acetyl | *O*-benzoyl | Euphorbiaproliferin D |

**Fig. S9.** Chemical Structures of myrsinane diterpenes present in *E. grantii.*

|  | | | | | | | | |
| --- | --- | --- | --- | --- | --- | --- | --- | --- |
| **#** | **R_1_** | **R_2_** | **R_3_** | **R_4_** | **R_5_** | **R_6_** | **R_7_** | **Compound name** |
| **Type A** | | | | | | | | |
| **13** | OH | OH | OH | H | H | H | H | Euphelionolide A |
|  | OH | OH | H | H | β-OH | H | H | Euphelionolide B |
|  | OH | OH | H | H | α-OH | H | H | Euphelionolide C |
|  | H | α-OH | H | H | H | OH | OH | Euphelionolide D |
|  | H | β-OH | H | H | H | OH | OH | Euphelionolide E |
|  | H | α-OH | OH | H | H | H | OH | Euphelionolide F |
|  | H | α-OH | H | OH | H | H | OH | Euphelionolide G |
| **Type B** | | | | | | | | |
| **13** | OH | OH | H | H | H | OH |  | Euphelionolide L |
| **Type B** | | | | | | | | |
| **22-23** | H | OH | OH | H | H | =O |  | Euphelionolide N |
| **Type A** | | | | | | | | |
| **22-23** | H | =O | H | OH | H | H | OH | Euphelionolide H |
|  | =O | OH | OH | H | H | H | H | Euphelionolide I |
| **Type A** | | | | | | | | |
| 26 | =O | OH | H | OH | H | H | OH | Euphelionolide J |
|  | =O | OH | H | H | H | OH | OH | Euphelionolide K |

**Fig. S10:** Chemical structures of ent-abietane diterpenes found in *E. grantii*.

**Fig. S11.** Chemical Structures of jolkinolide B.

**Fig. S12.** Chemical Structures of monoterpenoids found in *E. grantii*.

**Fig. S13.** Chemical Structures of sesquiterpenoids found in *E. grantii*.

**Fig. S14.** Chemical Structures of triterpenes found in *E. grantii*.
